# Btla signaling in conventional and regulatory lymphocytes coordinately tempers humoral immunity in the intestinal mucosa

**DOI:** 10.1016/j.celrep.2022.110553

**Published:** 2022-03-22

**Authors:** Caroline Stienne, Richard Virgen-Slane, Lisa Elmén, Marisol Veny, Sarah Huang, Jennifer Nguyen, Elizabeth Chappell, Mary Olivia Balmert, Jr-Wen Shui, Michelle A. Hurchla, Mitchell Kronenberg, Scott N. Peterson, Kenneth M. Murphy, Carl F. Ware, John R. šedý

**Affiliations:** 1 Immunity and Pathogenesis Program, Sanford Burnham Prebys Medical Discovery Institute, La Jolla, CA 92037, USA; 2 Tumor Microenvironment and Cancer Immunology Program, Sanford Burnham Prebys Medical Discovery Institute, La Jolla, CA 92037, USA; 3 La Jolla Institute for Immunology, La Jolla, CA 92037, USA; 4 Department of Pathology and Immunology, Washington University in Saint Louis School of Medicine, Saint Louis, MO 63110, USA; 5 Lead contact

## Abstract

The Btla inhibitory receptor limits innate and adaptive immune responses, both preventing the development of autoimmune disease and restraining anti-viral and anti-tumor responses. It remains unclear how the functions of Btla in diverse lymphocytes contribute to immunoregulation. Here, we show that Btla inhibits activation of genes regulating metabolism and cytokine signaling, including *Il6* and *Hif1a*, indicating a regulatory role in humoral immunity. Within mucosal Peyer’s patches, we find T-cell-expressed Btla-regulated Tfh cells, while Btla in T or B cells regulates GC B cell numbers. Treg-expressed Btla is required for cell-intrinsic Treg homeostasis that subsequently controls GC B cells. Loss of Btla in lymphocytes results in increased IgA bound to intestinal bacteria, correlating with altered microbial homeostasis and elevations in commensal and pathogenic bacteria. Together our studies provide important insights into how Btla functions as a checkpoint in diverse conventional and regulatory lymphocyte subsets to influence systemic immune responses.

## In brief

Stienne et al. investigate how BTLA and HVEM receptors control inflammatory and metabolic signaling in lymphocytes. A major function of these receptors in intestinal lymphoid tissue is to regulate mucosal antibody production. Ultimately, this system controls the composition of the intestinal microbiome, including pathogenic bacteria.

## Graphical abstract

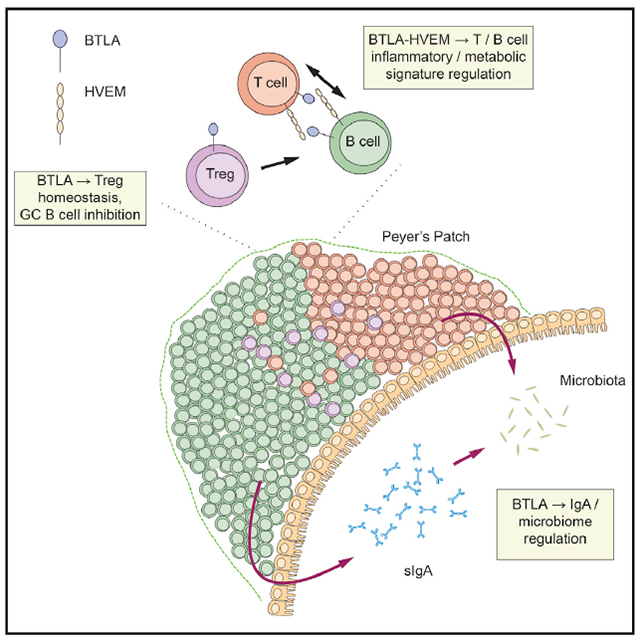

## INTRODUCTION

Checkpoint receptors, such as B and T lymphocyte attenuator (Btla), are widely expressed in lymphocyte and myeloid cells, where they function to regulate innate and adaptive immune responses and prevent the development of autoimmune disease (Schildberg et al., 2016; Ward-Kavanagh et al., 2016; Zhang and Vignali, 2016). Btla is activated by its ligand, herpesvirus entry mediator (Hvem, Tnfrsf14), which induces cytoplasmic recruitment of Shp-1 and Shp-2 tyrosine phosphatases that modulate signals activated by lymphocyte antigen receptors (Sedy and Ramezani-Rad, 2019). Phosphatase recruitment by Btla or the checkpoint protein programmed cell death protein-1 (PD-1, *Pdcd1*) inhibits T cell activation, providing evidence for targeting both receptors together in cancer immunotherapy (Celis-Gutierrez et al., 2019). Btla expression is high in B cells and relatively less in T follicular helper T (Tfh) cells, where it regulates Il21 signaling, indicating a role in regulating adaptive humoral responses (Crotty, 2011; Hurchla et al., 2005; Sedy et al., 2013). Aged *Btla*-deficient animals develop increased levels of systemic antibodies and spleen germinal center (GC) reactions, correlating with the development of autoimmune liver hepatitis (Oya et al., 2008). However, it was not clear how Btla specifically regulated T or B cells in humoral responses *in vivo* and whether Btla regulatory function was restricted to T cells. Additionally, the impact of Btla inhibition on the activation of gene expression in these lymphocytes was not clear.

Development of autoimmune disease has been linked to an imbalance or inappropriate control of commensal microbiota within the host intestine. *Pdcd1*-deficient animals showed enhanced GC responses in gut lymphoid tissues and elevated IgA associated with dysbiosis of host microbiota (Kawamoto et al., 2012). *Btla*-deficient animals were more susceptible to autoimmune disease in multiple models, including in experimental autoimmune encephalomyelitis (EAE) (Watanabe et al., 2003). Binding of Btla by its ligand Hvem or by an agonistic antibody inhibits T cell proliferation and effector function, limiting disease in experimental models (Albring et al., 2010; Bekiaris et al., 2013). Alterations in gut microflora in humans and mice are linked to multiple sclerosis or EAE disease (Berer et al., 2011, 2017; Lee et al., 2011). Commensal bacteria may amplify pro-inflammatory pathways, such as segmented filamentous bacteria (SFB) stimulation of T helper 17 (Th17) cells differentiation, or anti-inflammatory pathways through short-chain fatty acids (SCFAs) and tryptophan metabolites produced by species in *Bacteroides* and *Clostridium* clusters IV and XIVa (Brown et al., 2019). IgA in mucosal tissues coats intestinal bacteria, illustrating how the immune system interacts with and shapes the microbiome. The role of Btla in regulating IgA production and microbial homeostasis is not known.

The specific roles of diverse checkpoint proteins in lymphocytes and how Btla functions in diverse lymphocyte populations during immune responses remain to be defined. Here, we sought to compare autonomous Btla activity in T and B cells and to examine the impact of Btla signaling and loss of Btla expression in homeostatic control of immunity and the development of autoimmune disease. Thus, we used genetic models of conditional Btla deletion to identify the role of Btla in immune cell populations in lymphoid tissues that respond to and regulate host gut microflora, including conventional T cells, regulatory T cells, and B cells. Using these models, we also examined the functional impact of Btla regulation of mucosal immunity, including IgA production and regulation of microbial homeostasis. Together our studies provide important insights into how Btla functions as a checkpoint in conventional and regulatory lymphocyte subsets within the gut mucosa that may influence systemic immune responses.

## RESULTS

### Btla inhibits effector cell transcriptional signatures in T and B cells

**Figure 1.**
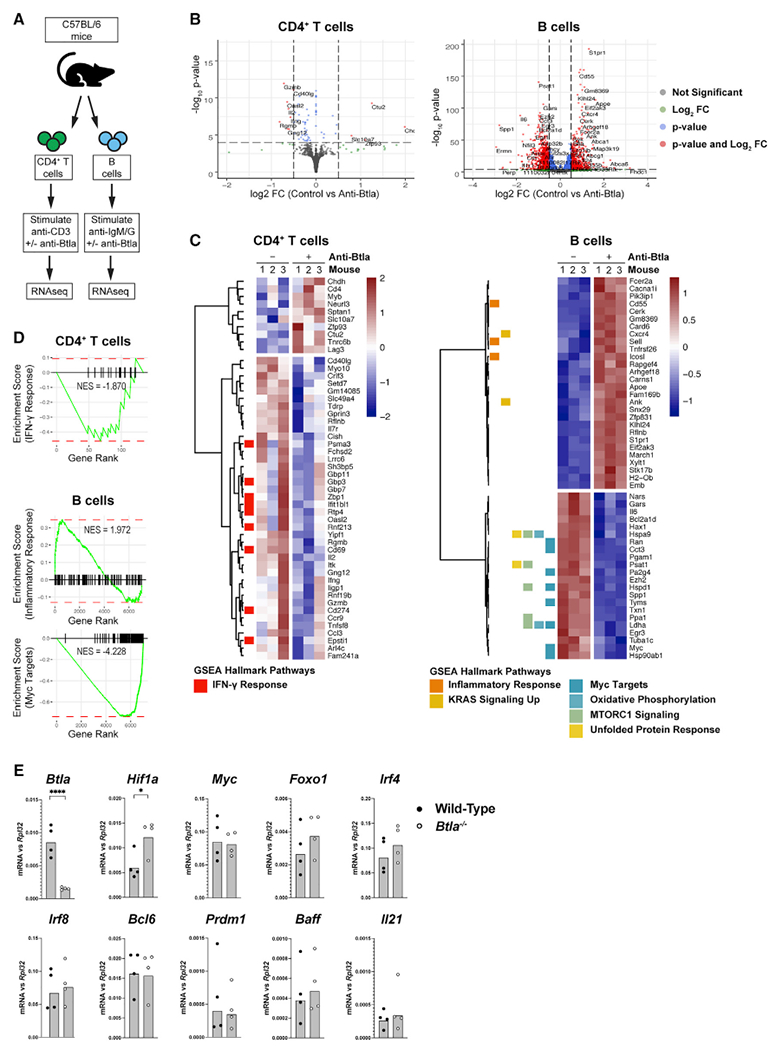
Anti-Btla inhibits inflammatory signaling in T and B cells (A) Setup for RNAseq experiment with C57BL/6 animals. (B) Volcano plots indicating genes significantly altered in anti-Btla-treated cells compared with control in anti-CD3-activated CD4^+^ T cells or anti-IgM/IgG activated. (C) Heatmaps of significantly altered genes in CD4^+^ T or B cells with GSEA hallmark pathways shown. (D) Hallmark GSEA pathways in CD4^+^ T and B cells. (E) Graphs of gene expression in germinal center B cells in SRBC-immunized wild-type or *Btla*^−/−^ hosts. Data analyzed using Student’s t test. *p < 0.05; ****p < 0.0001. Representative two experiments with n = 4 replicates for each condition. See also Figure S1.

We and others have previously found that the inhibitory receptor Btla inhibits activation of inflammatory signaling by diverse lymphocyte and myeloid cells, providing a brake to the development of autoimmune disease (Bekiaris et al., 2013; Ward-Kavanagh et al., 2016; Watanabe et al., 2003). However, the molecular mechanisms through which Btla receptors function in diverse cell types remain unknown. We sought to compare how Btla regulates gene transcription in T and B cells activated by their antigen receptors (Figure 1A). We used an anti-Btla monoclonal antibody (mAb) (clone 6A6) that binds Btla at its ligand-binding domain, resulting in blockade of interactions with endogenous Hvem and also in antibody-mediated activation of inhibitory signaling (Albring et al., 2010; Bekiaris et al., 2013; Hurchla et al., 2005). As expected, the transcriptional profiles regulated by Btla in T cell receptor (TCR)-stimulated primary CD4^+^ T cells and B cell receptor (BCR)-stimulated primary B cells were markedly different, with the number of genes impacted by anti-Btla in T cells (n = 133, adjusted p < 0.1) much less than in B cells (n = 7027, adjusted p < 0.1) (Figure 1B). In T cells, anti-Btla treatment resulted primarily in downregulation of transcripts, a majority of which are induced following TCR activation, including *Cd40lg, Il7r, Cish, Cd69, Il2, Itk, Ifng, Cd274* (PD-L1), and *Tnfsf8* (CD30L) (Figure 1C). Notably, CD40lg (CD154) was recently identified as a target of Btla inhibitory signaling that regulates GC responses (Mintz et al., 2019). Gene set enrichment analysis (GSEA) of the differentially expressed genes in T cells indicated a significant overall downregulation of the hallmark interferon gamma (IFN-γ) response gene set (normalized enrichment score [NES] = −1.870, Figures 1D and S1A). Interestingly, anti-Btla stimulation in T cells activated regulatory genes, including the *Lag3* inhibitory receptor and the transcription factor *Myb* that is required for differentiation of thymus-derived regulatory T (Tres) cells (Dias et al., 2017; Ruffo et al., 2019). In B cells, anti-Btla stimulation reduced the expression of BCR-induced transcripts associated with B cell differentiation and activation, including *Myc, Ezh2, Il6,* and *Ldha* (Figure 1C) (Beguelin et al., 2013; Luo et al., 2018). Globally, the predominant signatures associated with anti-Btla stimulation in B cells were downregulated genes within the hallmark Myc targets gene set (NES = −4.228) as well as genes associated with metabolism (oxidative phosphorylation, NES = −3.424; mammalian target of rapamycin complex 1 [mTORC1] signaling, NES = −3.419), while pathways upregulated by anti-Btla were not as significant (hallmark inflammatory response, NES = 1.972, Figures 1D and S1A). *Myc* and *Ezh2* both are required for intact progression of B cells through the GC reaction, indicating that this pathway is a major target of Btla inhibition.

To confirm Btla regulation of transcriptional pathways in lymphocytes, we examined how activating human BTLA with an agonist mAb (clone MIH26) regulated transcription in the absence of antigen receptor signaling both to identify BTLA-specific signals and to determine which signals required additional synergy (Figure S1B) (Otsuki et al., 2006). In both human CD4^+^ T and B cells, anti-BTLA stimulation inhibited expression of inflammatory genes associated with cytokine signaling, including a shared core set of genes (*CSF3, HIF1A, IL1A, IL1B, IL6,* and *PTGS2*) and confirmed regulation of type I interferon by BTLA in B cells (Figures S1C–S1E) (Sedy et al., 2017). Interestingly, BTLA regulated *IL6* expression in both human and mouse B cells regardless of antigen receptor stimulation. In order to confirm Btla regulation of GC-associated B cell transcripts, we examined levels of transcription factors in wild-type and *Btla*-deficient GC B cells purified from sheep red blood cell (SRBC)-immunized hosts (Figure S1F) (Milpied et al., 2018; Ramezani-Rad and Rickert, 2021; Singh et al., 2014). In *Btla*-deficient GC B cells, the levels of *Hif1a* were significantly elevated, consistent with a derepression of this factor (Figure 1E). Together, these data indicate that Btla broadly regulates inflammatory signaling in T and B cells, including metabolic reprogramming. Additionally, we find that a major target of Btla inhibition in both T and B cells following antigen receptor activation is the gene network that controls GC responses.

### T-cell-restricted Btla controls GC B cell expansion

**Figure 2.**
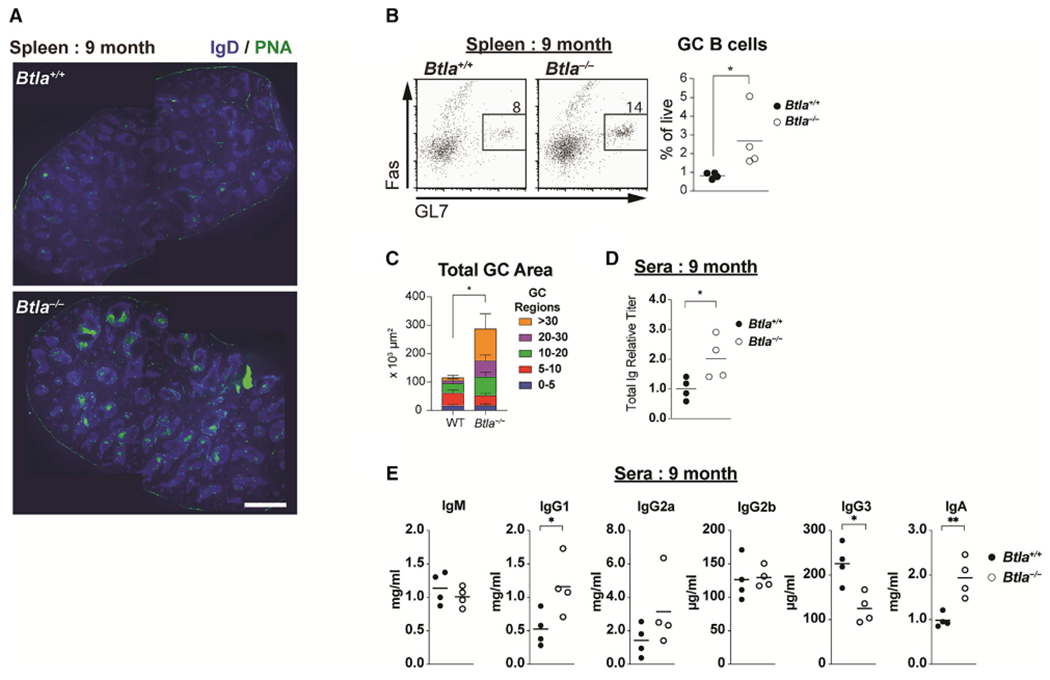
Btla regulates spontaneous GC reactions in aged animals (A) Spleen sections from 9-month-old wild-type or *Btla*^−/−^ animals stained with IgD and peanut agglutinin (PNA). Scale bar equals 1 mm. (B) Plots and graph of CD19^+^IgD^low^GL7^+^Fas^+^ spleen GC B cells in aged wild-type and *Btla*^−/−^ animals. (C) Stacked bar graph of total area of GC reactions in (A). (D and E) Graphs of total serum Ig (D) and isotype-specific antibody titers (E) in aged wild-type and *Btla*^−/−^ animals. Data analyzed using Student’s t test. Error bars indicate SD. *p < 0.05; **p < 0.01. Representative of four separate experiments with n = 4 replicates for each condition. See also Figures S3A–S3I.

A next-generation therapeutic mode is the activation of inhibitory receptors like BTLA to treat autoimmune disease (Paluch et al., 2018). However, interpretation of systemically administered Btla agonists is complicated by broad receptor expression. Thus, we used genetic models of lineage-specific deletion to determine the cellular requirements for Btla and how they may impact immunity (Figures S2A and S2B) (Seo et al., 2018; Ward-Kavanagh et al., 2016). Spontaneous autoreactive antibodies previously observed in aged *Btla*-deficient animals were associated with the development of autoimmunity (Oya et al., 2008). We confirmed an increased presence of spontaneous GC reactions in the spleens of *Btla*-deficient animals between 6 and 14 months of age compared with age-matched wild-type animals both by histological and flow cytometric analysis, with many visibly larger GC in *Btla*-deficient spleens (Figures 2A–2C and S3A–S3F). The combined area of all GC in *Btla*-deficient spleens mice was approximately 3-fold greater than in wild-type animals, with the increase entirely due to the presence of GC reactions that had grown to areas greater than 10^4^ μm^2^. The total antibody titer in *Btla*-deficient mice was approximately 2-fold greater than in wild-type, due to significant increases in immunoglobulin G (IgG) as well as in serum IgA (Figures 2D and 2E). In younger animals, we observed a trend for increased antibody titers to a model antigen in *Btla*−/− animals compared with wild-type animals following immunization, although this was not associated with changes to antibody affinity (Figures S3G–S3I).

**Figure 3.**
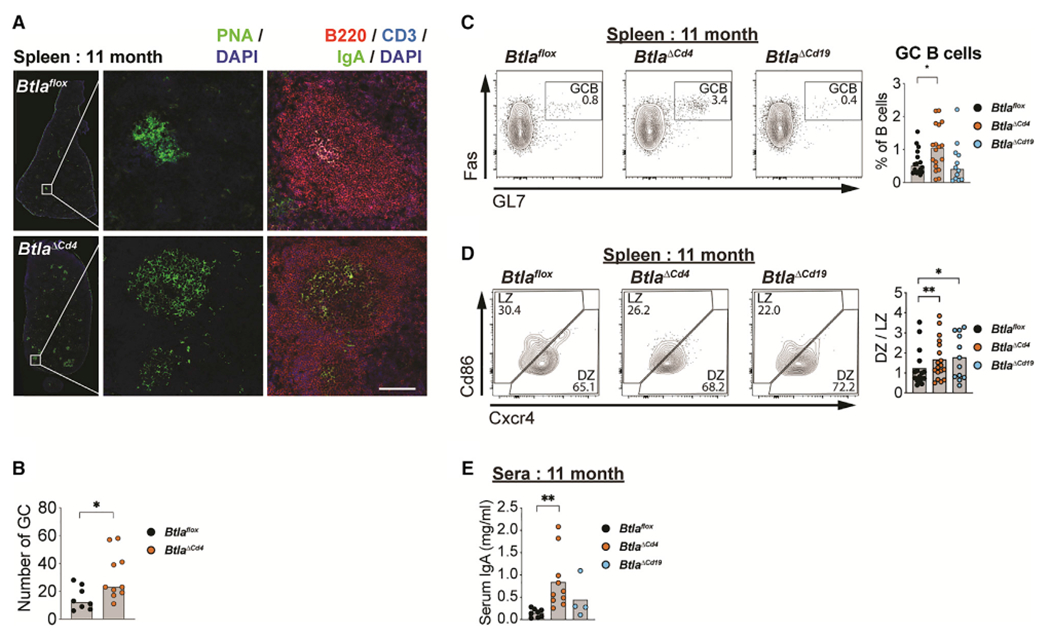
Btla expression in T cells limits serum IgA in aged animals (A) Spleen sections from 11-month-old *Btla*^*flox*^ and *Btla*^*ΔCd4*^ animals stained for PNA and DAPI or B220, CD3, IgA, and DAPI. Scale bar equals 100 μm. (B) Graph of number of PNA^+^ GC in (A). (C) Plots and graph of IgD^low^CD38^low^GL7^+^Fas^+^ GC B cells in CD19^+^ B cells. (D) Plots of Cxcr4^hi^CD86^lo^ DZ and Cxcr4^lo^CD86^hi^ LZ cells and graph of DZ/LZ cell ratio in spleens of aged *Btla*^*flox*^ and *Btla*^*ΔCd4*^ animals. (E) Graph of serum IgA in aged *Btla*^*flox*^, *Btla*^*ΔCd4*^, and *Btla*^*ΔCd19*^ animals. Data analyzed using multi-parameter linear modeling ANOVA. *p < 0.05; **p < 0.01. Representative of four separate experiments with n = 4 replicates for each condition. See also Figures S2 and S3J–S3M.

We next used *Btla^flox^* animals to determine the cellular basis for elevated splenic GC reactions in aged cohorts and to determine the immune cell subsets that regulated these reactions, including Tfh, Treg, T follicular regulatory (Tfr), and GC B cells (Figures 3A–3C, S2C–S2E, and S3J). We observed a significant increase in the number of GC in spleen histological sections and in the frequency of GC B cells in animals lacking *Btla* in T cells (*Btla*^ΔCd4^), compared with *Btla*^*flox*^ animals, and a trend for increased Tfh cells. Importantly, we also observed expression of IgA^+^ B cells in these GC reactions as well as increased IgA in the serum of *Btla*^*ΔCd4*^ animals, confirming regulation of IgA by T cell Btla (Figures 3A and 3E). Within the GC, B cells transit between the histologically defined and hypoxic light zone (LZ), in which B cells are stimulated by antigen-specific Tfh cells, and the normoxic dark zone (DZ) that is the primary site of B cell proliferation (Boothby and Rickert, 2017; Mesin et al., 2016; Victora et al., 2010). B cell transit between these areas is in part regulated by Hif1a that promotes glycolysis and is governed by O_2_ levels. Interestingly, we observed a significant increase in the DZ/LZ ratio estimated by the expression of Cxcr4 and Cd86 receptors in both the *Btla*^*ΔCd4*^ and *Btla*^*ΔCd19*^ strains compared with *Btla*^*flox*^ animals, potentially reflecting altered metabolic programming within the GC (Figure 3D). *Hvem* deletions in either T or B cells showed trends for increased GC B cells (*Tnfrsf14*^*ΔCd4*^, p = 0.111; *Tnfrsf14*^*ΔCd19*^, p = 0.059), indicating that expression of *Hvem* in both cell populations may contribute to the regulation of spontaneous GC reactions (Figures S3K–S3M). However, the impact of Hvem deletion was not as significant as deletion of Btla in T cells, indicating that Btla expression in T cells primarily regulates this response. Thus, Btla regulation of T cells restrains the development of spontaneous GC in peripheral lymphoid tissues, resulting in increased IgA production.

### Btla activates cell-intrinsic regulatory functions within mucosal GC

**Figure 4.**
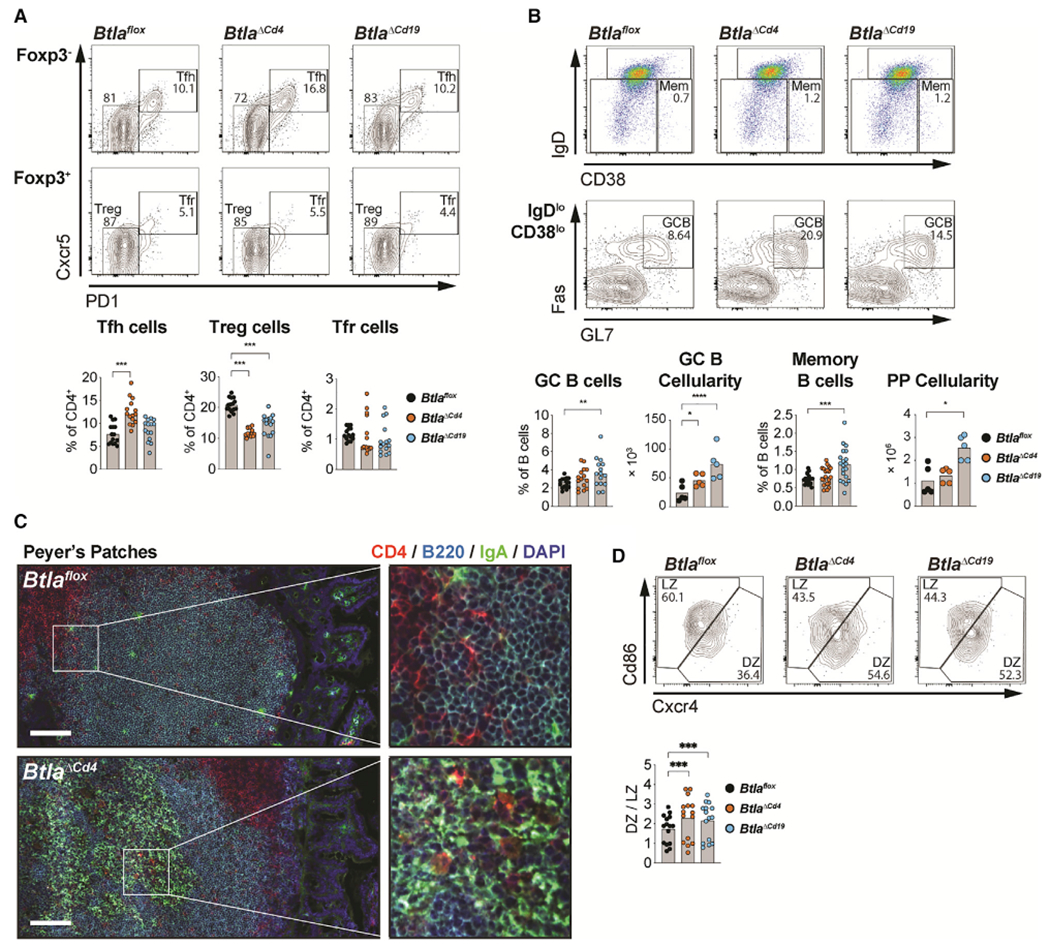
Btla expression in T and B cells regulates GC reactions in PPs (A and B) Plots and graphs of Foxp3^−^PD1^+^Cxcr5^+^ Tfh, Foxp3^+^PD1^−^Cxcr5^−^ Treg, and Foxp3^+^PD1^+^Cxcr5^+^ Tfr cells (A) and IgD^low^CD38^hi^ memory B cells, IgD^low^CD38^low^GL7^+^Fas^+^ GC B cells, GC B cell cellularity, and PP cellularity (B) in PPs of 8-week-old *Btla*^*flox*^, *Btla*^*ΔCd4*^, and *Btla*^*ΔCd19*^ animals. (C) PP sections from 8-week-old *Btla*^*flox*^ and *Btla*^*ΔCd4*^ animals stained with anti-CD4, anti-B220, anti-IgA, and DAPI. Scale bar equals 100 μm. (D) Plots of Cxcr4^hi^CD86^lo^ DZ and Cxcr4^lo^CD86^hi^ LZ cells and graph of DZ/LZ cell ratio in PPs of 8-week-old *Btla*^*flox*^, *Btla*^*ΔCd4*^, and *Btla*^*ΔCd19*^ animals. Data analyzed using multi-parameter linear modeling ANOVA. *p < 0.05; **p < 0.01; ***p < 0.001; ****p < 0.0001. Representative of three separate experiments with n = 4 replicates for each condition. See also Figures S4A–S4H.

Several studies have linked altered immune homeostasis and gut dysbiosis to the development of multiple sclerosis, which we had first shown was exacerbated in animals lacking *Btla* (Berer et al., 2011; Lee et al., 2011; Watanabe et al., 2003). Elevated serum IgA in *Btla*-deficient animals indicated a role for Btla inhibitory function in mucosal immune responses. Thus, we next examined how Btla regulated homeostasis of mucosal immune cell subsets and chronic GC within mucosal lymphoid tissues that form in response to commensal flora. Within the Peyer’s patches (PPs) of *Btla*^*ΔCd4*^ animals, we observed an increased frequency of Tfh cells and a reduced frequency of Treg cells as well as an increased cellularity of GC B cells compared with *Btla*^*flox*^ animals (Figures 4A and 4B). In sections of PPs from *Btla*^*ΔCd4*^ animals, we observed a greatly increased expression of B cell-associated mucosal IgA compared with *Btla*^*flox*^ animals, indicating elevated B cell function, as well as increased frequency of CD4^+^ cells within the B cell follicle (Figure 4C). In *Btla*^*ΔCd19*^ animals, we also observed a trend for increased frequency of Tfh cells and a significant reduction of Treg cell frequency, with an overall increase in Tfh numbers (Figure S4A). In *Btla*^*ΔCd19*^ animals, we also observed a significantly increased frequency of GC B cells that resulted in a 3-fold increase in the absolute numbers of GC B cells as well as a 2-fold increase in PPs cellularity compared with *Btla*^*flox*^ animals. In *Btla*^*ΔCd19*^ animals, the frequency of CD38^hi^ IgD^lo^ memory B cells was also significantly increased compared with *Btla*^*flox*^ animals, indicating increased output from GC reactions, as well as the percent and cellularity of IgA^+^ GC B cells, indicating a B cell intrinsic role for Btla in regulating antibody production (Figure S4B) (Bemark et al., 2016).

We confirmed elevated *Ldha, Il6,* and *Hif1a* in non-mitogen-activated B cells and a trend for increased Btla-regulated transcripts in non-mitogen-activated T cells from PPs of *Btla*-deficient animals compared with wild-type, consistent with deregulation of metabolic pathways (Figure S4C). Notably, Ldha and Hif1a activate glycolytic signaling, while interleukin-6 (Il-6) regulates B cell expansion and differentiation (Boothby and Rickert, 2017; Hunter and Jones, 2015; O’Neill et al., 2016). In T cells, Il-6 can function with Il-21 to stimulate Tfh cell differentiation and with transforming growth factor β (TGF-β) to inhibit Treg differentiation while promoting Th17 cell differentiation. In T cells we did not observe differences in Bcl6 or Il-21 protein expression that may influence Tfh cell frequencies (Figure S4D and S4E). In both *Btla*^*ΔCd4*^ and *Btla*^*ΔCd19*^ GC B cells, we observed an increased ratio of DZ/LZ cells compared with *Btla*^*flox*^ GC B cells that may reflect an altered metabolic setpoint (Figure 4D). However, despite elevations in metabolic pathway genes, we did not observe increased bromodeoxyuridine (BrdU) uptake in PP lymphocytes from *Btla*-deficient animal strains (Figures S4F and S4G). We next compared Btla and Hvem in mucosal and peripheral lymphocytes to identify unique expression patterns that may be linked to tissue-specific phenotypes (Figure S4H). Btla expression is elevated in Treg cells but reduced in B cells in PPs compared with spleen. In contrast, Hvem is reduced in Treg cells in PPs and also reduced in non-naive B cell subsets. Additionally, the Btla/Hvem ratio is increased in Tconv and Treg cells, increasing accessibility to Btla ligand-induced activation and potentially explaining increased sensitivity of PP T cells to Btla activation (Cheung et al., 2009a). Interestingly, reduction of Treg cells in the absence of Btla in either T or B cells raised the possibility that GC homeostasis was in part maintained via cell-cell cross-regulation by Treg cells and that Btla functioned to maintain this T cell subset.

Peripheral differentiation of Treg cells in the intestine occurs in response to environmental stimuli, which are presented indirectly via dendritic cells (DCs) that express TGF-β and IL-10 (Brown et al., 2019). We thus examined whether expression of Btla in DCs could regulate GC lymphocyte populations in the PPs, because these cells continually survey the mucosa and present antigens to T and B cells as well as secrete cytokines to promote T cell differentiation (Brown et al., 2019). Additionally, *Btla*-expressing DCs were shown to regulate Treg cell differentiation in the EAE model (Jones et al., 2016). However, in *Btla*^*ΔZbtb46*^ animals that lack Btla in conventional DCs, we did not observe any changes in Tfh, Treg, Tfr, or GC B cells compared with *Btla*^*flox*^ animals (Figure S4I).

### Hvem expressed in T and B cells regulates mucosal GC reactions

**Figure 5.**
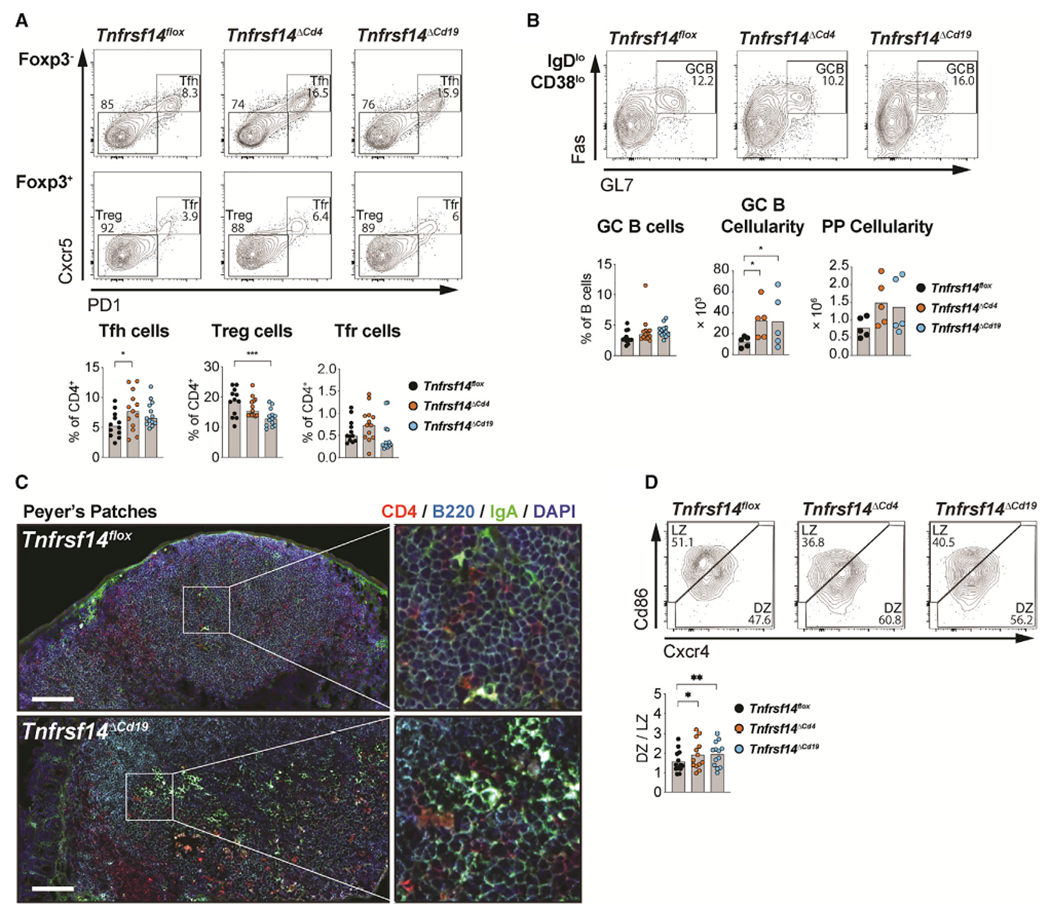
Hvem expression in T and B cells regulates GC reactions in PPs (A and B) Plots and graphs of Foxp3^−^PD1^+^Cxcr5^+^ Tfh, Foxp3^+^PD1^−^Cxcr5^−^ Treg, and Foxp3^+^PD1^+^Cxcr5^+^ Tfr cells (A) and IgD^low^CD38^low^GL7^+^Fas^+^ GC B cells, GC B cellularity, and PP cellularity in PPs of 8-week-old *Tnfrsf14*^*flox*^, *Tnfrsf14*^*ΔCd4*^, and *Tnfrsf14*^*ΔCd19*^ animals (B). (C) PP sections from 8-week-old *Tnfrsf14*^*flox*^ and *Tnfrsf14*^*ΔCd19*^ animals stained with anti-CD4, anti-B220, anti-IgA, and DAPI. Scale bar equals 100 μm. (D) Plots of Cxcr4^hi^CD86^lo^ DZ and Cxcr4^lo^CD86^hi^ LZ cells and graph of DZ/LZ cell ratio in PPs of 8-week-old *Tnfrsf14*^*flox*^, *Tnfrsf14*^*ΔCd4*^, and *Tnfrsf14*^*ΔCd19*^ animals. Data analyzed using multi-parameter linear modeling ANOVA. *p < 0.05; **p < 0.01; ***p < 0.001. Representative of three separate experiments with n = 4 replicates for each condition. See also Figures S4I and S4J.

We next asked which Hvem-expressing cells could engage Btla within PsP to limit GC responses using a model of conditional *Tnfrsf14* deletion. In *Tnfrsf14*^*ΔCd4*^, we observed increased frequency of Tfh cells compared with controls and a similar trend for Tfh cell numbers, while in *Tnfrsf14*^*ΔCd19*^ animals, we observed decreased frequency of Treg cells compared with *Tnfrsf14*^*flox*^ (Figures 5A and S4J). *Tnfrsf14*^*ΔCd19*^ animals also showed a trend for increased Tfh cells, and *Tnfrsf14*^*ΔCd4*^ also showed a trend for decreased Treg cells. However, these data indicate that Btla in Tfh cells may be predominantly engaged by Hvem in T cells, while Btla in Treg cells may be predominantly engaged by Hvem in B cells. Additionally, we observed a 3-fold increase in the absolute cellularity of GC B cells in *Tnfrsf14*^*ΔCd4*^ and *Tnfrsf14*^*ΔCd19*^ animals that phenocopied increases observed in *Btla*^*ΔCd19*^ animals (Figure 5B). We also identified increased IgA-positive B cells within in the PPs isolated from *Tnfrsf14*^*ΔCd19*^ animals compared with *Tnfrsf14*^*flox*^ animals and found more frequent CD4^+^ cells in the B cell follicle, similar to *Btla*^*ΔCd4*^ animals (Figure 5C). Finally, in GC B cells from both *Tnfrsf14*^*ΔCd4*^ and *Tnfrsf14*^*ΔCd19*^ animals, the DZ/LZ ratio was increased compared with GC B cells from *Tnfrsf14*^*flox*^ animals, similar to cell-specific Btla ablation (Figure 5D). These data illustrate a complex network of interactions controlled by Btla and Hvem in distinct lymphocyte subsets. We interpret these data to mean that T and B cell Hvem engages Btla in Tfh and Treg cells, respectively, and both cells can engage Btla in B cells to regulate GC B cell homeostasis. However, we cannot exclude the possibility that the effects of Hvem deletion may be dependent on the activities of its other ligands (Sedy and Ramezani-Rad, 2019).

### Btla activates cell-intrinsic expansion of regulatory T cells to limit GC B cells

**Figure 6.**
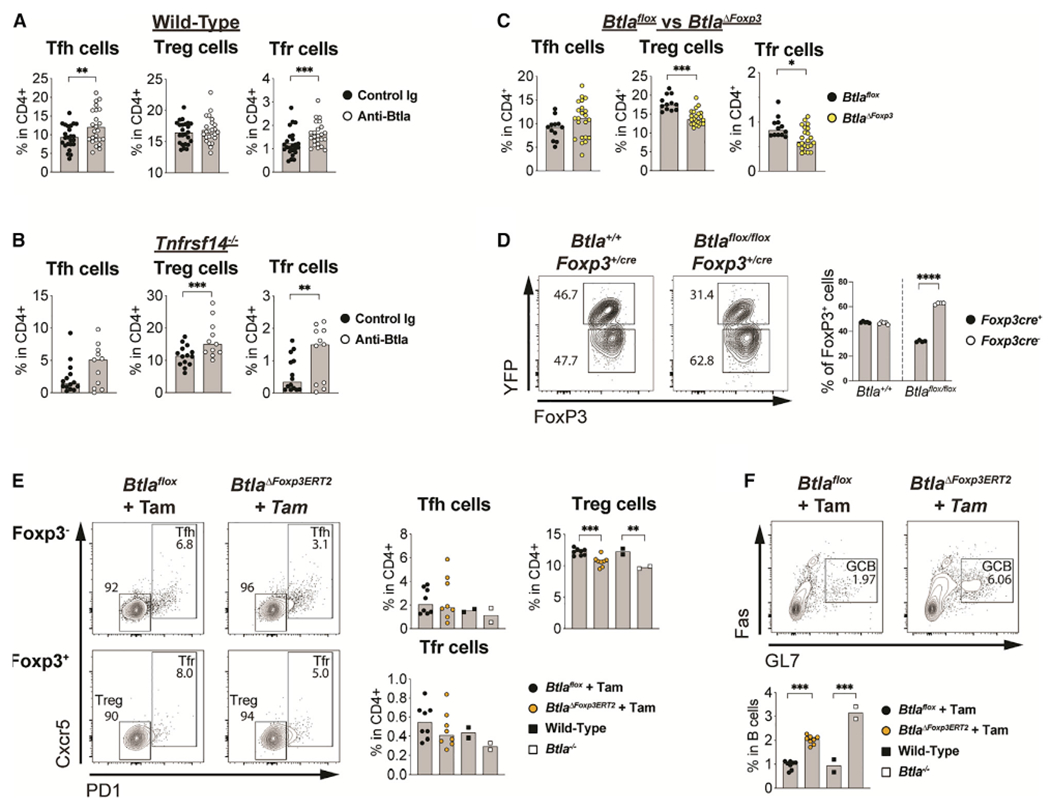
Cell-intrinsic maintenance of Treg cell homeostasis and function by Btla in PPs (A–C) Graphs of T cell subsets in PPs of control or anti-Btla-treated wild-type (A), *Tnfrsfl14*^−/−^ (B) animals, or of untreated *Btla*^*flox*^ and *Btla*^*ΔFoxp3*^ animals (C). (D) Plots and graph of FoxP3^+^YFP^+/−^ Treg cells in PPs of untreated wild-type *Foxp3*^*+/cre*^ and *Btla*^*flox/flox*^*Foxp3*^*+/cre*^ animals. (E and F) Plots and graphs of Foxp3^−^PD1^+^Cxcr5^+^ Tfh, Foxp3^+^PD1^−^Cxcr5^−^ Treg, and Foxp3^+^PD1^+^Cxcr5^+^ Tfr cells (E) and IgD^low^CD38^low^GL7^+^Fas^+^ GC B cells (F) in PPs of untreated wild-type, *Btla*^−/−^, and tamoxifen-treated *Btla*^*flox*^ and *Btla*^*ΔFoxp3ERT2*^ animals. Tam, yamoxifen. Data analyzed using multi-parameter linear modeling ANOVA. *p <0.05; **p<0.01; ***p < 0.001; ****p < 0.0001. Representative of six(wild-type) experiments, with at least n = 5 replicates for each condition; two (*Tnfrsf14*^−/−^) experiments, with at least n = 7 replicates for each condition in antibody treatment experiments; and four (constitutive) experiments, with at least n = 3 replicates for each condition and two (induced) Treg cells *Btla* deletion experiments, with at least n = 8 replicates for each condition. See also Figures S5 and S6.

The increases in Tfh and GC B cell frequencies observed in *Btla*- or *Tnfrsf14*-deficient genetic models are consistent with Btla inhibitory activity in these cells. However, the reduced frequency of Treg cells indicated that a costimulatory signal downstream of Btla may be dominant in this subset (Murphy and Murphy, 2010). We first examined whether agonistic Btla mAb (clone 6A6) could supplement endogenous Hvem to regulate homeostasis of lymphocyte subsets, including Treg cells. Surprisingly, anti-Btla consistently increased the frequency of both Tfh and Tfr cells within PPs, although there was no change in conventional Treg cells (Figures 6A and S5A). We further examined the impact of anti-Btla in the absence of Hvem, since the *in vivo* efficacy of Btla activation was previously shown to be mitigated by the expression of its endogenous ligand (Cheung et al., 2009a; Steinberg et al., 2008). In *Tnfrsf14*-deficient animals, we observed a significant increase in both Treg and Tfr subsets, consistent with a supportive function for Btla in these regulatory populations (Figures 6B and S5B). Administration of anti-Btla also increased Treg cell frequencies in the spleens of unchallenged wild-type and *Tnfrsf14*-deficient animals (Figures S5C and S5D). The frequency of Foxp3^+^ cells in the thymus was not changed, indicating a direct effect on peripheral Treg cells (Figure S5E). Anti-Btla did not affect the frequency of GC B cells in the absence of Hvem, although the population of memory B cells in the PP swas significantly decreased in these animals, indicating reduced GC output (Figures S5F and S5G). Thus, activation of Btla appeared to promote Treg cell expansion, as was previously suggested in a model of graft-versus-host disease (Albring et al., 2010).

To confirm a cell-intrinsic supportive function of Btla in Treg cells, we analyzed animals containing deletion of *Btla* in Foxp3^+^ cells (*Btla*^*ΔFoxp3*^). In the PPs of *Btla*^*ΔFoxp3*^ animals, we observed a decrease of CD4^+^ Foxp3^+^ Treg and Tfr cells compared with *Btla*^*flox*^ animals, with no significant change in Tfh or GC B cells (Figures 6C and S5H–S5J). We next analyzed Foxp3-cre^+/−^ heterozygous female animals, within which half the cells express the Cre gene due to X-linked silencing and in which we visualized its expression because this protein is fused to yellow fluorescent protein (YFP) (Rubtsov et al., 2008). In *Foxp3^+/Yfp-Cre^* animals, we observed the expected 1:1 ratio of YFP^+^ to YFP^−^ FoxP3^+^ Treg cells (Figure 6D). However, in *Btla*^*flox/flox*^*Foxp3^+/Yfp-Cre^* animals, among FoxP3^+^ Treg cells, there were half as many YFP^+^ Treg cells compared with YFP^−^ Tregs, indicating that loss of Btla was detrimental to Treg cell homeostasis when in competition with Btla-sufficient cells. Interpretation of *Btla* deletion in Foxp3^+^ T cells was complicated due to the early instability of CD4^+^ T cell polarization (Brown et al., 2019). We circumvented confounding developmental factors by using a genetic model of induced *Btla* deletion. In *Btla*^*ΔFoxp3ERT2*^ animals treated with tamoxifen, we again observed a reduced frequency of Treg cells in the PPs compared with tamoxifen-treated *Btla*^*flox*^ animals, similar to reductions seen in *Btla*^*ΔFoxp3*^ animals and similar to untreated *Btla^−/−^* animals (Figures 6E, S6A, and S6B). We also now observed a 2-fold increase in GC B cells in the PPs of tamoxifen-treated *Btla*^*ΔFoxp3ERT2*^ animals compared with tamoxifen-treated *Btla*^*flox*^ animals that was associated with an increase in the GC B cell DZ/LZ ratio (Figure 6F). Treg cells in the PPs of tamoxifen-treated *Btla*^*ΔFoxp3ERT2*^ animals did not show a reduction of Ctla4, Il-10, and Il-1β effector proteins that may contribute to increased GC B cell frequencies (Figures S6C–S6E). We next examined whether Btla activation in Treg cells directly activated homeostatic signaling through Myc because it is essential for maintenance of Treg cells *in vivo* and because it is a primary target of Btla regulation in B cells (Saravia et al., 2020). In SRBC-immunized animals treated with Btla mAb, we detected a specific increase in Myc expression in FoxP3^+^ cells compared with control Ig-treated animals (Figure S6F). In contrast, we did not observe changes in Myc expression in FoxP3^−^ cells. Together, these data indicate a unique cell-intrinsic function for Btla in maintenance of Treg cells, and through Treg cells an indirect regulation of GC B cell homeostasis.

### Btla regulates mucosal antibody production and microbial homeostasis

**Figure 7.**
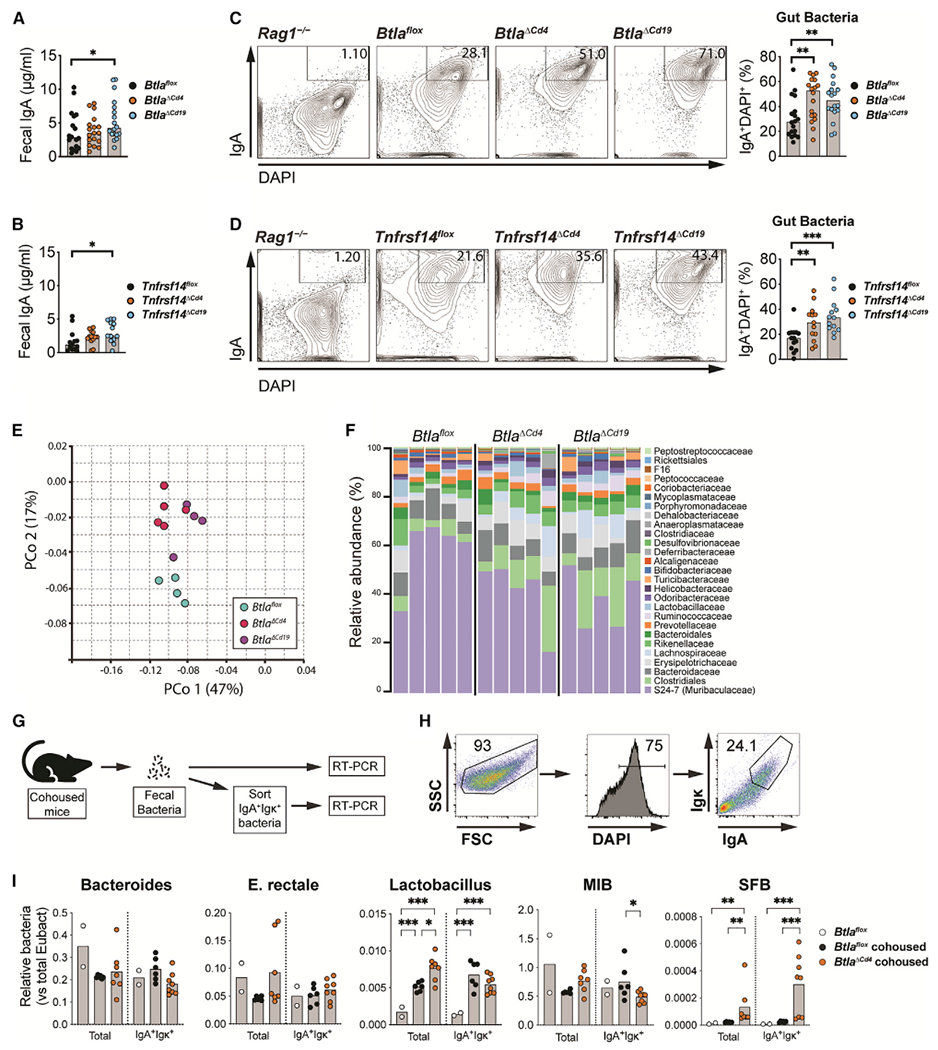
Lymphocyte expressed Btla and Hvem regulate microbial homeostasis (A and B) Graphs of IgA in fecal pellets of *Btla*^*flox*^, *Btla*^*ΔCd4*^, and *Btla*^*ΔCd19*^ animals (A), and *Tnfrsf14*^*flox*^, *Tnfrsf14*^*ΔCd4*^, and *Tnfrsf14*^*ΔCd19*^ animals (B). (C and D) Plots and graphs of IgA^+^DAPI^+^ bacteria in fecal pellets of *Rag1*^−/−^, *Btla*^*flox*^, *Btla*^*ΔCd4*^, and *Btla*^*ΔCd19*^ animals (C), and *Rag1*^−/−^, *Tnfrsf14*^*flox*^, *Tnfrsf14*^*ΔCd4*^, and *Tnfrsf14*^*ΔCd19*^ animals (D). (E and F) PcoA plot (E) and graph of microbial β diversity (F) in fecal pellets of *Btla*^*flox*^, *Btla*^*ΔCd4*^, and *Btla*^*ΔCd19*^ animals. (G–I) Experimental setup (G), plots of sorting strategy (H), and qRT-PCR graphs of total and IgA^+^Igκ^+^ bacteria species (I) in cohoused *Btla*^*flox*^ and *Btla*^*ΔCd4*^ animals. MIB, mouse intestinal *Bacteroides*; SFB, segmented filamentous bacteria; data analyzed using multi-parameter linear modeling ANOVA. *p < 0.05; **p < 0.01; ***p < 0.001. Representative of five (*Btla*^*flox*^, *Btla*^*ΔCd4*^, and *Btla*^*ΔCd19*^) experiments with at least n = 4 replicates for each condition, three (*Tnfrsf14*^*flox*^, *Tnfrsf14*^*ΔCd4*^ and *Tnfrsf14*^*ΔCd19*^) experiment swith at least n = 4 replicates for each condition, and two (cohoused *Btla*^*flox*^ and *Btla*^*ΔCd4*^) experiments with at least n = 4 replicates for each condition. See also Figure S7.

We next sought to determine whether Btla regulated mucosal GC functions, including IgA production, and whether IgA impacted microbial community homeostasis in the intestine. Compared with *Btla*^*flox*^ and *Tnfrsf14*^*flox*^ animals, we observed elevated levels of IgA within the fecal material of *Btla*^*ΔCd19*^ and *Tnfrsf14*^*ΔCd19*^ animals and a trend for increased IgA in *Btla*^*ΔCd4*^ and *Tnfrsf14*^*ΔCd4*^ animals (Figures 7A and 7B). In these young animals, we observed elevations in serum IgA within *Btla*^*ΔCd4*^ and *Tnfrsf14*^*ΔCd4*^ animals compared with control strains, although the increases were small compared with those observed in aged animals (Figures 3E, S7A, and S7B). A large proportion of IgA was bound to bacteria in all immunocompetent strains (Figures 7C and 7D). However, the proportion of IgA bound to bacteria was increased in both *Btla*^*ΔCd4*^ and *Btla*^*ΔCd19*^ cohorts compared with *Btla*^*flox*^ animals and in both *Tnfrsf14*^*ΔCd4*^ and *Tnfrsf14*^*ΔCd19*^ cohorts compared with *Tnfrsf14*^*flox*^ animals. Mucosal IgA exhibits diverse functions, including coating effacing bacteria and preventing pathologic infection, in order to control microbial homeostasis at mucosal surfaces (Brown et al., 2019). Analysis of β diversity of microbial communities revealed that *Btla*^*ΔCd4*^ and *Btla*^*ΔCd19*^ strains both clustered together away from the *Btla*^*flox*^ strain in the principal coordinate analysis (PCoA) (Figure 7E). The microbiota of both *Btla*^*ΔCd4*^ and *Btla*^*ΔCd19*^ strains were characterized by an expansion of a number of bacterial species, including several *Clostridium* spp. that increased in both strains (Figures 7F, S7C, and S7D). In both mouse strains the top scoring taxa was *Alistipes putridinis*, a propionate-producing bacterium, and both also displayed a reduced relative abundance of *Parasutterella excrementihominis*. While the differentially abundant taxa in *Btla*^*ΔCd4*^ and *Btla*^*Δ*^*Cd19* animals was otherwise distinct, both featured a number of butyrate-producing bacteria. We next determined the abundance of specific bacteria through amplification of species-specific qRT-PCR (Barman et al., 2008). Notably, compared with *Btla*^*flox*^ animals, fecal samples from *Btla*^*ΔCd19*^ animals showed significant elevation in Th17-cell-promoting SFB normally resident in the small intestine (Figure S7E). We further examined whether IgA in *Btla*-deficient strains directly influenced gut microbiota through analysis of specific bacteria in the IgA^+^ fraction (Figures 7G and 7H). In cohoused littermates of *Btla*^*ΔCd4*^ animals compared with *Btla*^*flox*^ animals, we observed elevations in both *Lactobacillus* and in SFB (Figure 7I). Notably, while increases in *Lactobacillus* were partly attributable to environment, increased SFB were entirely linked to genetic background. Additionally, SFB were enriched in the IgA^+^ fraction of *Btla*^*ΔCd4*^ animals, magnifying differences between this strain and control animals. We additionally examined whether administration of agonistic Btla mAb could also shape microbial homeostasis, and thus potentially impact local and systemic immune responses. Analysis of β diversity of microbial communities showed a clustering of animals dosed with Btla mAb discrete from animals dosed with control Ig, as displayed in PcoA, although the observed changes were less striking than in the *Btla*-deficient strains (Figure S7F). Agonistic Btla mAb treatment was also associated with an expansion of several species of *Clostridium* spp. as well as *Bacteroides* spp. (Figures S7H–S7I). Additionally, the bacteria species with the greatest expansion following Btla mAb treatment included *Clostridium* clusters IV and XIVa species *C. asparagiforme, O. valericigenes, O. ruminantium*, and *F. butyricus*, all known butyrate producers, and notably did not overlap with any of the bacteria taxa over-represented in *Btla*^*Δ*^*Cd19* animals (Iino et al., 2007; Lagkouvardos et al., 2016; Lee et al., 2013; Mohan et al., 2006). Butyrate is known to promote Treg cell differentiation and anti-Btla may additionally influence Treg cell frequency through its effects on the microbiome (Arpaia et al., 2013). Although we did not previously observe differences in GC B cell frequencies in *Btla*^*ΔFoxp3*^ animals, we examined whether Btla in Treg cells could regulate microbial diversity. However, in *Btla*^*ΔFoxp3*^ animals, we did not observe a significant difference in the global diversity of fecal bacteria compared with *Btla*^*flox*^ animals (Figures S7J and S7K). Together, these data illustrate how lymphocyte-expressed *Btla* directly regulates mucosal IgA that shapes the microbiome. Our findings thus provide evidence for how Btla functions locally in lymphocytes to regulate signaling and effector function, thus influencing mucosal immune responses and cross-talk between the host and microbiota.

## DISCUSSION

Here, we examined the function of the Btla inhibitory receptor in diverse lymphocytes. Our study identifies gene inhibition signatures in T and B cells that control lymphocyte collaboration within the GC. Older animals with Btla-deficient T cells showed increased GC reactions and GC B cells that expressed IgA in the spleen compared with wild-type. Younger animals with Btla-deficient T cells showed increased B-cell-expressed IgA in ongoing PP GC reactions responsive to host microbiota. This increase paralleled elevated Tfh and GC B cell numbers and reduced Treg cell frequency in PPs. We show that Btla promotes expansion of Treg cells and that the frequency of GC B cells is increased with acute deletion of Btla in Treg cells. Loss of Btla in either T or B cells resulted in elevated IgA coating of bacteria and skewing of the microbial diversity. Thus, Btla functions in multiple lymphocyte compartments to control GC reactions and IgA production in the intestinal mucosa, ultimately regulating homeostasis of host microbiota.

Our bioinformatic analysis indicates that metabolic signaling is a major pathway targeted by Btla in B cells through regulation of genes, such as Hif1a and Myc. Abundant Hif1a expressed in GC B cells is responsive to hypoxic conditions, activating glycolytic signaling (Boothby and Rickert, 2017; O’Neill et al., 2016). Myc controls activation of glycolysis and entry into cell cycle and is required for T and B cell activation (Pollizzi and Powell, 2014; Wang et al., 2011). In B cells, Myc is required for Pax5-mediated differentiation, and, in GC B cells, Myc activates AP4 expression to promote IL-21 signaling and progression to the DZ (Calado et al., 2012; de Alboran et al., 2001; Dominguez-Sola et al., 2012; Mesin et al., 2016; Vallespinos et al., 2011). Interestingly, Hif1a inhibits the activity of Myc, indicating that Btla may be required for proper GC B cell selection, differentiation, and antibody production. We reasoned that in GC B cells, Btla targets BCR signaling, regulating expression of metabolism pathway genes and cell-cycle entry, thus restraining B cell maturation (Luo et al., 2018; Vendel et al., 2009). Btla-regulated gene transcription in B cells was robust compared with T cells, due to higher Btla expression in B cells that is accessible to exogenous ligands compared with ligand inaccessible Btla-Hvem cocomplexes (Hurchla et al., 2005; Sedy et al., 2013, 2017). Our data confirm regulation of Tfh-cell-specific factors, including Il6CD40lg t, that together with IL-21 stimulate Tfh cell differentiation, and CD40lg that was shown to be downregulated by Btla in antigen-stimulated T cells (Hunter and Jones, 2015; Mintz et al., 2019). Our data are consistent with a previous report showing increased *in vitro* Tfh cell differentiation of *Btla^−/−^* T cells stimulated with Il-6 (Kashiwakuma et al., 2010). Our analysis is also consistent with a study of checkpoint protein targets in anti-CD3-activated T cells that identified Btla-regulated genes, including *Il7r, Cish, Gzmb, Cd274,* and *Cd40lg,* and also a costimulatory component to Btla activity (Wakamatsu et al., 2013). Together, these data indicate a critical role for Myc and Il-6 as targets of Btla in regulating mucosal immune responses.

Btla promotes cell survival *in vivo*, including in cell transfer models of graft-versus-host disease and inflammatory bowel disease (Murphy and Murphy, 2010). We propose that Treg cell Btla may activate costimulatory signaling, potentially mediated through a YDND motif in the cytoplasmic domain that is required for optimal CD8^+^ T cell proliferation and IL-2 production and may promote Myc expression (Ritthipichai et al., 2017). In T cells, Myc is required for activation-induced metabolic reprograming but is also required for Treg cell development (Saravia et al., 2020; Wang et al., 2011). Btla inhibition of HIF1A is consistent with Treg cell differentiation because Hif1a promotes Th17 cell differentiation, skewing T cells away from the Treg cell fate (Shi et al., 2011). Additionally, microbial-produced butyrate has been shown to inhibit HIF1A nuclear translocation (Zgouras et al., 2003). Thus, BTLA may promote Treg cells through inhibition of HIF1A and through microbiome regulation. Our observations that Btla signaling expands the Treg cell subset *in vivo* and that Btla is required for cell-intrinsic Treg cell homeostasis provide evidence that one aspect of Btla function is to promote Treg cell activity *in vivo*. Btla inhibition of *IL6* expression in human and mouse T and B cells may also allow TGF-β-induced Treg cell differentiation. While Btla controls the development of conventional Treg and Tfr cells in GC reactions, the suppressive function of *Btla*-deficient Treg cells was not different from wild-type Treg cells (Tao et al., 2008). Thus, we reasoned that the role of Btla may be to stimulate differentiation of Treg cells with specialized activity.

We found that Btla supports Treg cells in the PPs to regulate the frequency of GC B cells. In contrast, we did not observe a clear role for Btla in Treg cell inhibition of Tfh cells. The mechanism of Treg cell control of B cell activation continues to be an area of active investigation, potentially involving Ctla-4 blocking of CD80/CD86-mediated T cell costimulation, secretion of tolerogenic cytokines, such as IL-10, or granzyme B-mediated cytolysis (Sage and Sharpe, 2015; Wing et al., 2018). A recent report of acute deletion of Tfr cells demonstrated that these cells regulate GC responses (Clement et al., 2019). Acute deletion of Btla may uniquely affect peripheral Treg cells, including Tfr cells in PP GC reactions, prior to the emergence of *Btla*-deficient thymic-derived Treg cells that develop with an altered TCR repertoire. T and B cell-specific Btla deletion was partly phenocopied by T and B cell deletions of *Tnfrsf14*. B cell Hvem activates Btla in T cells to limit activation of GC microenvironment-stimulating chemokines and development of B cell lymphomagenesis (Boice et al., 2016; Mintz et al., 2019). Btla activates Hvem receptors and downstream nuclear factor κB (NF-κB) survival signaling (Cheung et al., 2009b; Sedy and Ramezani-Rad, 2019; Ward-Kavanagh et al., 2016). Thus, it remains possible that cell-intrinsic Hvem signaling may additionally impact mucosal GC responses.

Mucosal IgA regulates intestinal microflora directly through neutralization, immune exclusion from mucosal epithelium, alteration of bacterial gene expression, and trapping within the mucous layer, thus influencing global interactions between species and shifting the makeup of the microbial community (Sutherland et al., 2016). In Btla-deficient animals, alterations in SCFAs and tryptophan metabolite-producing commensal bacteria may influence Treg cell differentiation and thus immune homeostasis (Brown et al., 2019). Btla deficiency also resulted in elevations in Th17 cells, promoting SFB. Activating Btla receptors using agonist mAb-induced changes to the microbiome that we hypothesize would be further magnified in animals lacking Hvem. *Pdcd1*-deficient animals showed impaired control of IgA, microbial dysbiosis, and strain-dependent autoimmune disease susceptibility, similar to *Btla*-deficient strains (Kawamoto et al., 2012; Zhang and Vignali, 2016). However, PD-1 specifically regulated Tfh cells and did not influence Treg cells development (Kawamoto et al., 2012; Zhang et al., 2016). Thus, the unique function for Btla in stimulating Treg distinguishes it from PD-1 *in vivo*.

Together, these results underscore the significance of the BTLA pathway in regulating immunity and highlight the potential to target this protein in human disease using agonist or antagonist therapeutics. Reports continue to emerge linking BTLA polymorphisms to autoimmune disease, development of cancer, or viral infection (Sedy et al., 2014; Sedy and Ramezani-Rad, 2019). A better understanding of how BTLA functions in regulatory T cells will shed light on immune dysfunction in human disease. While the specific role of BTLA in each of these pathologies remains to be determined, harnessing the BTLA-HVEM network will provide a useful addition to the clinician’s toolbox.

### Limitations of the study

Our study focuses primarily on analysis of the role of BTLA and HVEM in GC responses, both in aged animals and in the PPs of the intestine gastrointestinal tract. Data from aged animals represent stochastic development of spontaneous GCs within the spleen. We were not able to determine the time of GC development a priori and thus these data represent a snapshot of GCs at different stages of development. Analyses of PP immune responses may not be generally applicable to all mucosal surfaces due to the unique nature of diverse tissues. In our analyses of immune responses in the gut mucosa, we were not able to determine specific responses due to the difficulty and ongoing research into bacteria antigen reactivity. Analyses of BTLA and HVEM in follicular Treg cells was not addressed due to the lack of specific genetic models for this subset.

## STAR★METHODS

### RESOURCE AVAILABILITY

#### Lead contact

Further information and requests for resources and reagents should be directed to and will be fulfilled by the lead contact, Carl F. Ware (cware@sbpdiscovery.org).

#### Materials availability

*Btla*^*flox*^ and *Tnfrsf14*^*flox*^ genetic strains are available for research purposes upon signing a materials transfer agreement. *Btla*^*−/−*^ and *Cre*-expressing strains are available from Jackson Labs. Anti-mouse Btla (clone 6A6) and control hamster Ig are available from Bio X Cell. Anti-human BTLA (clone MIH26) is available from ThermoFisher.

#### Data and code availability

RNAseq, Nanostring, and microbiome 16S sequencing data are available upon request and are deposited at NCBI GEO and are publically available as of the date of publication. Accession numbers are listed in the key resources table. Microscopy data reported in this paper will be shared by the lead contact upon request. This paper does not report original code. Any additional information required to reanalyze data reported in this paper is available from the lead contact upon request.

### EXPERIMENTAL MODEL AND SUBJECT DETAILS

#### *In vivo* animal studies

All experiments were approved by the Sanford Burnham Prebys IACUC. Mice were bred to a C57BL/6J background that were refreshed with animals from The Jackson Laboratory and housed SBP Animal Facility unless otherwise noted. In all experiments animals between 8 to 12 weeks of age from both sexes were assigned to experimental groups unless otherwise indicated. Both *Btla*^*flox*^ and the previously described *Tnfrsf14*^*flox*^ animals were crossed at least ten generations onto the C57BL/6 background (Seo et al., 2018). *Btla*^*−/−*^ (Jackson Labs), 129SvEv (Taconic), and BALB/c (Jackson Labs) mice were bred in our facility. *Btla*^*−/−*^ mice (Watanabe et al., 2003) were backcrossed to C57BL/6 and BALB/c for nine generations each and were subsequently crossed onto the DO11.10 TCR-transgenic background (Murphy et al., 1990). *Cd4*^*cre*^ and *Cd19*^*cre*^ strains were bred as previously described (Lee et al., 2001; Rickert et al., 1997). *Foxp3*^*cre*^*, Foxp3*^*ERT2cre*^ and *Zbtb46*^*cre*^ strains were obtained from Jackson Labs. In experiments using *in vivo* antibody administration to unchallenged animals, 100 μg of agonistic Btla mAb (clone 6A6) or control hamster Ig was injected intra-peritoneally to cohoused wild-type animals twice per week for two weeks. In experiments using *in vivo* antibody administration to SRBC-immunized animals, 400 μg of control hamster Ig or agonistic Btla mAb (clone 6A6) was injected intraperitoneally once to co-housed wild-type animals. In experiments using tamoxifen-induced cre-mediated deletion of *Btla*, animals were injected intraperitoneally on three consecutive days with 1.5 mg of tamoxifen (Sigma). For cohousing experiments with *Btla*^*flox*^ and *Btla*^*ΔCd4*^ animals, littermates from heterozygous breeding pairs were genotyped and housed together from birth throughout all experiments.

#### Btla gene targeting and generation of flox animals

An 18.1 kb targeting vector containing a DTA selection cassette, a frt-flanked neo cassette, and two loxP sites flanking Btla exon 4 and 5 was generated as shown (Figure S2A). The vector was electroporated into 129 embryonic stem (ES) cells for homologous recombination and drug-resistant clones were identified and screened by Southern blot using a probe annealing upstream of exon 3. Confirmed ES cells were microinjected into blastocysts and then transferred to pseudopregnant female mice to generate chimera. Germline transmission was obtained by crossing to wild-type C57BL/6 animals. Conditional deletion of Btla genes was confirmed by surface staining of Btla in specific cell lineages depending on the Cre transgenic mice used for the analysis. *Btla*^*flox-neo/flox-neo*^ mice were generated using 129 ES cells by the UC San Diego Health Sciences transgenic mouse core facility.

#### Purification of human lymphocytes

Deidentified fresh human blood was collected from healthy donors greater than 18 years of age and of mixed gender giving written informed consent at the Scripps Research Institute Normal Blood Donor Service, and all handling was approved by the Sanford Burnham Prebys Medical Discovery Institute Internal Review Board. Samples were mixed 1:1 with PBS and overlaid onto Ficoll (GE Healthcare) for density gradient centrifugation. PBMC were isolated from buffy coats and washed twice with PBS. Human B cells and CD4^+^ T cells were further purified using EasySep B cell enrichment or CD4^+^ T cell enrichment kits (Stemcell Technologies).

### METHOD DETAILS

#### Bioinformatic analysis of purified lymphocytes

For mouse lymphocyte transcriptional analysis, spleens, inguinal and brachial lymph nodes from C57BL/6 animals were harvested, disaggregated, and RBC were lysed with ACK lysis buffer. Mouse B cells or CD4^+^ T cells were further purified using EasySep B cell enrichment or CD4^+^ T cell enrichment kits (Stemcell Technologies). Purified CD4^+^ T cells or B cells were stimulated with anti-CD3 (clone 2C11) oranti-IgM/IgG (polyclonal, Jackson Immunoresearch) respectively, with or without anti-Btla (6A6, Bio X Cell) adsorbed to aldehyde/sulfate latex microspheres produced as previously described or control (Thermo Fisher Scientific) for 18 h at 37°C. (Hurchla et al., 2005; Sedy et al., 2017). Total RNA was prepared from stimulated B cells and T cells using RNeasy mini kit (Qiagen) and submitted to the Sanford Burnham Prebys Genomics Core for RNAseq processing and analysis. Samples were hybridized onto Illu-mina chips and analyzed using a HiSeq 1500 instrument (Illumina). Transcripts were quantified from raw fastq files using *Salmon*, and data were analyzed in R v4.0.0 using *DESeq2* (Love et al., 2014; Patro et al., 2017; Zhu et al., 2019). Data was visualized using *EnhancedVolcano* and *pheatmap*; gene enrichment using hallmark gene sets was performed using *fgsea*; bulk data was managed using the *Hotgenes* package (Blighe et al., 2020; Kolde, 2018; Korotkevitch et al., 2019; Liberzon et al., 2015; Virgen-Slane, 2020).

For human lymphocyte transcriptional analysis, purified cultures of human B cells or T cells were stimulated with anti-BTLA (MIH26) or control Ig adsorbed to aldehyde/sulfate latex microspheres produced as previously described and/or 10 U/ml IFN (Thermo Fisher Scientific, R & D Systems) for 6 h at 37°C. (Otsuki et al., 2006; Sedy et al., 2017). Cells were harvested in RLT buffer, immediately hybridized onto Human Inflammation v2 Panel chips, and processed with the nCounter instrument (Nanostring). Preliminary data analysis was performed using nSolver 4.0 software (Nanostring) and differential expression and statistical analysis was performed using the *NanoStringDiff* package in R v4.0.0 (Wang et al., 2016). Data was visualized using *pheatmap*; gene annotation and meta-analysis was performed using Metascape (Zhou et al., 2019).

#### GC B cell isolation and qRT-PCR analysis

GC B cells were induced in wild-type and *Btla*^*−/−*^ animals immunized with 10% Sheep Red Blood Cells (SRBC, Colorado Serum Company) in PBS as previously described (Cato et al., 2011). At day 8 following immunization spleens were harvested, disaggregated, and RBC lysed with ACK lysis buffer. Splenocytes were stained with bio-anti-CD43, bio-anti-CD11c, and bio-anti-IgD, and GC B cells were purified using Miltenyi anti-biotin microbeads (Miltenyi). Total RNA was prepared from purified B cells using the Qiagen RNeasy mini kit (Qiagen), and RNA was reverse-transcribed into cDNA using the iScript™ cDNA synthesis kit (BioRad) according to the manufacturer’s instructions. Relative expression of GC reaction transcripts was detected using SYBR Green Master Mix (Bio-Rad). Reactions were carried out in clear 384 well plates using an ABI® 7900HT Real-Time PCR System (Thermo Fisher Scientific). Relative expression was determined compared to *L32* and calculated as follows: 2^−ΔCt^, where Ct = cycle number, ΔCt = Ct(target gene) – Ct(*L32*). Oligonucleotide sequences are listed in Table S1.

#### Flow cytometry

To identify GC lymphocyte subsets, PP or spleens were dissected and harvested into single cell suspensions by centrifugation at 1800 rpm for 2 minutes at 4°C followed by staining with a fixable viability stain (Live/Dead Fixable Aqua Cell Stain Kit, ThermoFisher) for 10 minutes on ice in PBS. Surface antigens were then stained in FACS buffer for 1 hour on ice. For intracellular staining, after surface staining, cells were fixed using the Foxp3 transcription factor staining kit Fixation/Permeabilization buffer (ThermoFisher) for 20 minutes on ice. For transcription factor staining cells were then stained in the Permeabilization buffer for 25 minutes on ice followed by washing twice in PBS. All samples were washed in FACS buffer and analyzed. All samples were acquired using a LSRFortessa X-20 or on a FACSCalibur flow cytometer and FACSDiva software (BD Biosciences). Data were analyzed using FlowJo software (BD Biosciences). Lymphocytes were stained with mixtures of the following antibodies at a 1:400 dilution unless otherwise specified to identify T cell and B cell subsets, and their expressed transcription factors: CD95-FITC (Biolegend), B220-Percp-Cy5.5 (eBioscience), CXCR4-BV510 (BD Biosciences), CD86-BV605 (BD Biosciences), CD45-BV650 (BD Bioscience), GL7-Alexa Fluor647 (BioLegend), CD38-Alexa Fluor700 (ThermoFisher), TCRb-APC-Cy7 (BioLegend), IgA-PE (ThermoFisher), IgD-PE-Cy7 (BioLegend), CD19-BV711 (BD Bioscience), CD4-Alexa Fluor700 (BioLegend), CD62L-FITC (BioLegend), CD44-Percp-Cy5.5 (ThermoFisher), PD1-PE-Cy7 (BioLegend, 1:200 dilution), CXCR5-PE (ThermoFisher, 1:100 dilution), Foxp3-PB (BioLegend, 1:200) and Bcl6-APC (ThermoFisher, 1:200). In some experiments cells were stained with anti-IgD-PE (ThermoFisher), anti-GL7-FITC (BioLegend), anti-Fas-PE-Cy7 (BD Biosciences), and CD19-allophycocyanin (ThermoFisher), and PNA-Alexa 488 (ThermoFisher). The non-competing anti-Btla clone 8F4-PE (Biolegend) was used to analyze Btla levels in lymphocytes (del Rio et al., 2010; Hurchla et al., 2005; Truong et al., 2009). The anti-Hvem clone LH1-PE (ThermoFisher) that does not block Btla binding was used to analyze Hvem levels in lymphocytes.

#### Histological analysis

For immunofluorescence analysis, dissected spleens or PP were frozen in OCT, and sections were stained with a 1:100 dilution of anti-IgD-FITC (BD Biosciences) plus a 1:200 dilution of PNA-biotin (Vector Laboratories), or with anti-CD4-PE (ThermoFisher) plus anti-B220-APC (ThermoFisher) plus anti-IgA-Alexa488 (ThermoFisher), or with anti-CD3-PE (ThermoFisher).

#### Antibody ELISA

Mouse serum antibody titers were determined by ELISA using the Southern Biotechnology clonotyping/HRP kit for IgG subclass-specific ELISA (Southern Biotechnology Associates). Mouse IgA was analyzed using the Mouse IgA Ready-SET-Go! ELISA kit (ThermoFisher).

#### Microbiome sequencing and analysis

Mouse fecal pellets were collected, frozen on dry ice and stored at −80°C until processing. Bacterial DNA was extracted with QIAmp Fast DNA Stool Mini Kit (Qiagen) according to manufacturer’s instructions, with the addition of 5 minutes of glass bead-beating in lysis buffer AL, using Mini-Beadbeater-16 (Biospec Products). Library preparation was performed according to Illumina’s instructions for bacterial 16S ribosomal DNA gene sequencing, with PCR amplification of the V3-V4 region using the primer pair listed in Table S1. PCR products were purified at each step using the QIAquick 96-PCR Clean up kit (Qiagen). Libraries were quantified by QPCR using KAPA Library Quantification Kit for Illumina platforms (KAPA Biosystems). Sequencing was performed by the Institute for Genomic Medicine, UCSD (La Jolla CA) on the MiSeq instrument (Illumina). Raw data resulting from paired-end sequencing (250 bp) was processed and analyzed with the CLC Microbial Genomics Module (Qiagen). BLAST against the NCBI 16S Bacteria and Archaea database was used to assign taxonomy at genus and species level. Analysis of metagenomic abundance data was also done with LEfSe (Segata et al., 2011).

Fecal pellets were additionally processed for QRT-PCR analysis using the Fast DNA Stool Mini Kit (Qiagen). Aliquots of bacterial DNA were amplified using published primers for total Eubacteria, *Bacteroides, Eubacterium rectale/Clostridium coccoides, Lactobacillus/Lactococcus*, Mouse intestinal *Bacteroides*, and Segmented filamentous bacteria (Barman et al., 2008). QRT-PCR products were amplified using iTaq Sybr Green (Bio-Rad) on a CFX Real Time PCR Instrument (Bio-Rad).

### QUANTIFICATION AND STATISTICAL ANALYSIS

Unless otherwise indicated, in each set of experiments data from all biological replicates were analyzed together for significance using the linear modeling function in R v4.0.0 where each replicate was included as a distinct term in the formula (*response* ~ *replicate* + *term*). Data was plotted using Graphpad Prism 9.

## Supplementary Material

1

## Figures and Tables

**Table:** KEY RESOURCES TABLE

REAGENT or RESOURCE	SOURCE	IDENTIFIER
Antibodies		

Anti-mouse Btla clone 6A6	Bio X Cell	BE0132; RRID:AB_10949299
Hamster Ig isotype control	Bio X Cell	BE0091; RRID:AB_1107773
Anti-human BTLA clone MIH26	ThermoFisher	16-5979-82; RRID:AB_469173
Anti-mouse CD3 clone 145-2c11	ThermoFisher	16-0031-82; RRID:AB_468847
Anti-mouse IgM/IgG	Jackson Immunoresearch	115-005-068; RRID:AB_2338453
Anti-CD43 biotin	BD Biosciences	553269; RRID:AB_2255226
Anti-CD11c biotin	BD Biosciences	553800; RRID:AB_395059
Anti-IgD biotin	ThermoFisher	555777; RRID:AB_396112
Anti-CD95 FITC	Biolegend	152606; RRID:AB_2632901
Anti-B220 PerCP-Cy5.5	Biolegend	103236; RRID:AB_893354
Anti-CXCR4 BV510	BD Biosciences	563468; RRID:AB_2738225
Anti-CD86 BV605	BD Biosciences	743213; RRID:AB_2741349
Anti-CD45 BV650	BD Biosciences	563410; RRID:AB_2738189
Anti-GL7 Alexa Fluor 647	BioLegend	144606; RRID:AB_2562185
Anti-CD38 Alexa Fluor 700	ThermoFisher	102742; RID:AB_2890672
Anti-TCRb APC-Cy7	BioLegend	109220; RRID:AB_893624
Anti-IgA PE	ThermoFisher	12-4204-82; RRID:AB_465917
Anti-IgD PE-Cy7	BioLegend	405720; RRID:AB_2561876
Anti-CD19 BV711	BD Biosciences	563157; RRID:AB_2738035
Anti-CD4 Alexa Fluor 700	BioLegend	100430; RRID:AB_493699
Anti-CD62L FITC	BioLegend	104406; RRID:AB_313093
Anti-CD44 PerCP-Cy5.5	ThermoFisher	45-0441-82; RRID:AB_925746
Anti-PD-1 PE-Cy7	BioLegend	135208; RRID:AB_2159184
Anti-CXCR5 PE	ThermoFisher	12-7185-82; RRID:AB_11217882
Anti-Foxp3 Pacific Blue	BioLegend	126410; RRID:AB_2105047
Anti-Bcl6 APC	ThermoFisher	17-5453-82; RRID:AB_2573214
Anti-IgD PE	ThermoFisher	12-5993-82; RRID:AB_466113
Anti-GL7 FITC	BioLegend	144604; RRID:AB_2561697
Anti-Fas PE-Cy7	BD Biosciences	557653; RRID:AB_396768
Anti-CD19 APC	ThermoFisher	17-0191-82; RRID:AB_469358
PNA Alexa-488	ThermoFisher	L21409; RRID:AB_2315178
Anti-Btla clone 8F4 PE	BioLegend	134804; RRID:AB_1731884
Anti-Hvem clone LH1 PE	ThermoFisher	12-5962-80; RRID:AB_953628
Anti-IgD FITC	BD Biosciences	553439; RRID:AB_394859
PNA-biotin	Vector Labs	B-1075-5; RRID:AB_2336252
Anti-IgA FITC	ThermoFisher	11-4204-82; RRID:AB_465221
Anti-CD4 PE	ThermoFisher	12-0041-82; RRID:AB_465506
Anti-B220 APC	ThermoFisher	17-0452-82; RRID:AB_469395
Anti-CD3 PE	ThermoFisher	12-0031-82; RRID:AB_465496

Chemicals, peptides, and recombinant proteins		

Tamoxifen	Sigma-Aldrich	T5648
Ficoll	Avantor	95021-205
Tissue-Tek OCT	Sakura	4583
DAPI	Vector Labs	H-1200-10

Critical commercial assays		

EasySep Human B cell isolation kit	STEMCELL Technologies	17954
EasySep Human CD4+ T cell Isolation kit	STEMCELL Technologies	17952
EasySep Mouse B cell isolation kit	STEMCELL Technologies	19854
EasySep Mouse CD4+ T cell Isolation kit	STEMCELL Technologies	19852
RNeasy mini kit	Qiagen	74104
Anti-biotin microbeads	Miltenyi	130-090-485
iScript cDNA synthesis kit	Bio-Rad	1708891BUN
SYBR Green Master Mix	Bio-Rad	1725274
Live/Dead Fixable Aqua Cel Stain kit	ThermoFisher	L34957
FoxP3 transcription factor staining kit	ThermoFisher	00-5523-00
ELISA cloning/HRP kit	Southern Biotechnology	5300-05
Mouse IgA ELISA kit	ThermoFisher	EMIGA
QIAmo Fast DNA Stool kit	Qiagen	51604
QIAquick 96-PCR Clean Uo kit	Qiagen	28104
KAPA Library Quantification Kit	Roche	07960140001
iTaq Sybr Green	Bio-Rad	1725121

Deposited data		

RNAseq of mouse T and B cells	This paper	NCBI GEO: GSE196204
Nanostring of human T and B cells	This paper	NCBI GEO: GSE194314
Microbiome data	This paper	NCBI GEO: GSE195890

Experimental models: Organisms/strains		

*Btla^flox^*	This paper	N/A
*Tnfrsf14^flox^*	Seo et al., 2018	N/A
*Btla^−/−^*	The Jackson Laboratory	006353
129SvEv	Taconic	129S6
BALB/c	The Jackson Laboratory	000651
DO11.10	Murphy et al., 1990	N/A
*Cd4^cre^*	Lee et al., 2001	N/A
*Cd19^cre^*	Rickert et al., 1997	N/A
*Foxp3^cre^*	The Jackson Laboratory	016959
*Foxp3^ERT2cre^*	The Jackson Laboratory	016961
*Zbtb46^cre^*	The Jackson Laboratory	028538

Oligonucleotides		

See Table S1 for all oligonucleotide	N/A	N/A
sequences		

Software and algorithms		

ImageJ	Schneider et al., 2012	N/A
Prism 9	GraphPad	N/A
R v4.0.0	Team, 2021	https://www.R-project.org/
DESeq2	Love et al., 2014	N/A
Salmon	Patro et al., 2017	N/A
EnhancedVolcano	Blighe et al., 2020	N/A
Pheatmap	Kolde, 2018	N/A
Fgsea	Korotkevitch et al., 2019	N/A
Hotgenes	Virgen-Slane, 2020	N/A
nSolver 4.0	Nanostring	N/A
NanoStringDiff	Wang et al., 2016	N/A
Metascape	Zhou et al., 2019	N/A
FACSdiva	BD Biosciences	N/A
FlowJo	BD Biosciences	N/A
CLC Genomics Workbench	Qiagen	N/A
LEfSe	Segata et al., 2011	N/A

Other		

Sheep Red Blood Cells	Colorado Serum Company	31102
Aldehyde/Latex Beads 4% w/v 4 μm	ThermoFisher	A37304

## Highlights

- BTLA controls inflammatory and metabolic signatures in both T and B cells
- BTLA and HVEM regulate germinal center homeostasis in mucosal Peyer’s patches
- BTLA is required for homeostasis of regulatory T cells in the mucosa
- The BTLA-HVEM network controls gut microbiome homeostasis
