# Supplementary material for: Btla signaling in conventional and regulatory lymphocytes coordinately tempers humoral immunity in the intestinal mucosa
Source: Cell Rep. Author manuscript; Available in PMC 2022 Apr 22. (PMC9032671; doi:10.1016/j.celrep.2022.110553)
Supplement: 1 [file NIHMS1791869-supplement-1.pdf]

Cell Reports, Volume *38*

Supplemental information

Btla signaling in conventional and regulatory lymphocytes coordinately tempers humoral immunity in the intestinal mucosa

Caroline Stienne, Richard Virgen-Slane, Lisa Elmén, Marisol Veny, Sarah Huang, Jennifer Nguyen, Elizabeth Chappell, Mary Olivia Balmert, Jr-Wen Shui, Michelle A. Hurchla, Mitchell Kronenberg, Scott N. Peterson, Kenneth M. Murphy, Carl F. Ware, and John R. Šedý

Supplemental

Supplemental Figures

Figure S1

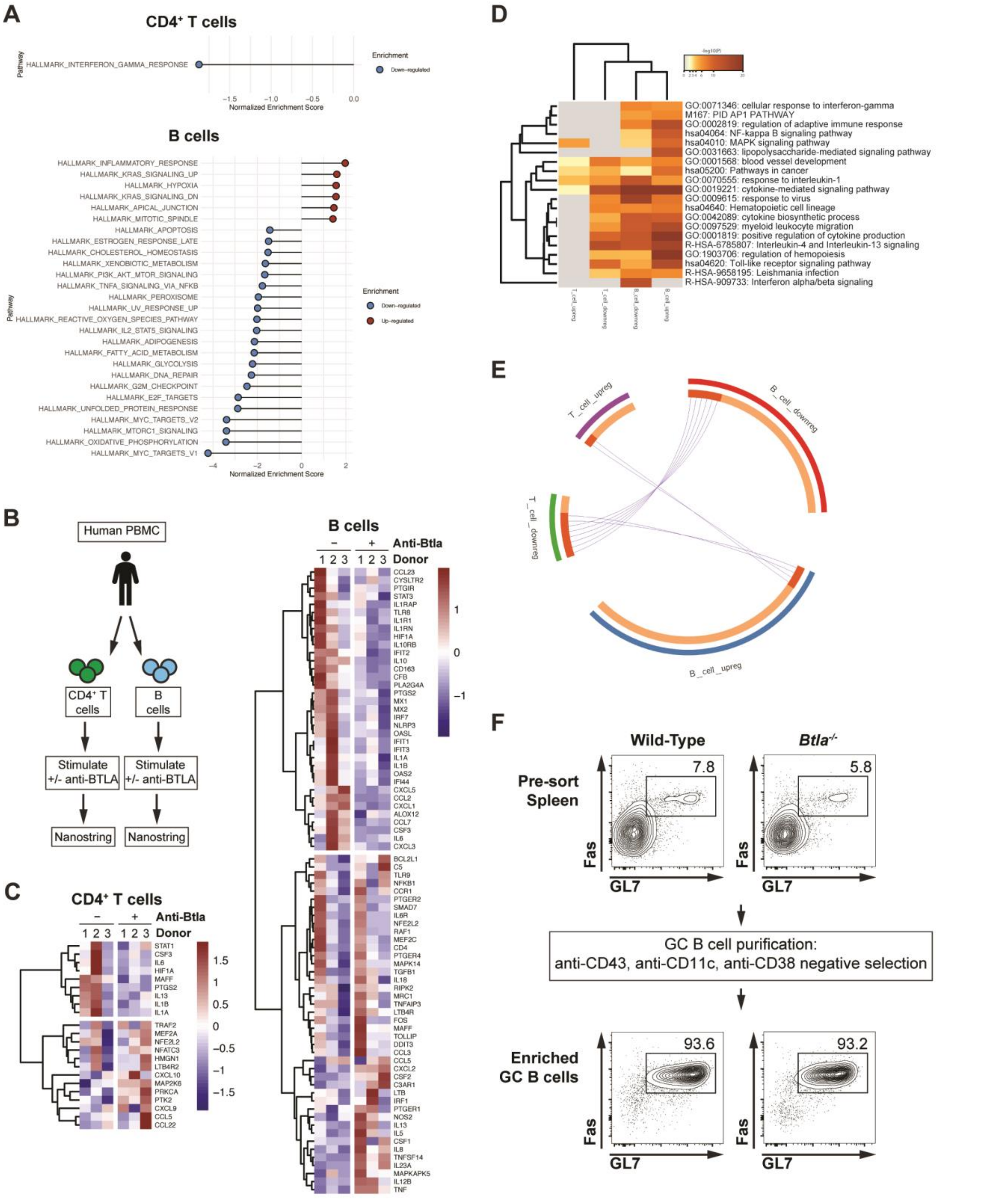

Figure S1. Hallmark gene signatures in mouse T and B cell RNAseq and human T and B

cell Nanostring. Related to main **Figure 1**. Graph of all Hallmark GS pathways and their Normalized Enrichment Scores identified in $CD4^+$ mouse T cells or mouse B cells. **B.** Setup for Nanostring experiment with human PBMC. **C.** Heatmaps indicating significantly altered genes in $CD4^+$ human T cells (left) or B cells (right) ($p < 0.05$). **D.** Enriched ontology clusters of downregulated genes in T cells (T_cell_downreg) and B cells (B_cell_downreg), and upregulated genes in T cells (T_cell_upreg) and B cells (B_cell_upreg). **E.** Circos plot of genes overlapping between T_cell_downreg/B_cell_downreg (*CSF3*, *HIF1A*, *IL1A*, *IL1B*, *IL6*, *PTGS2*), T_cell_upreg/B_cell_upreg (*NFE2L2*, *CCL5*), and T_cell_downreg/B_cell_upreg (*IL13*, *MAFF*). **F**. Strategy for enrichment of GC B cells from SRBC immunized animals. Flow plots show purity of GC B cells prior (top) and after (bottom) purification.

Figure S2

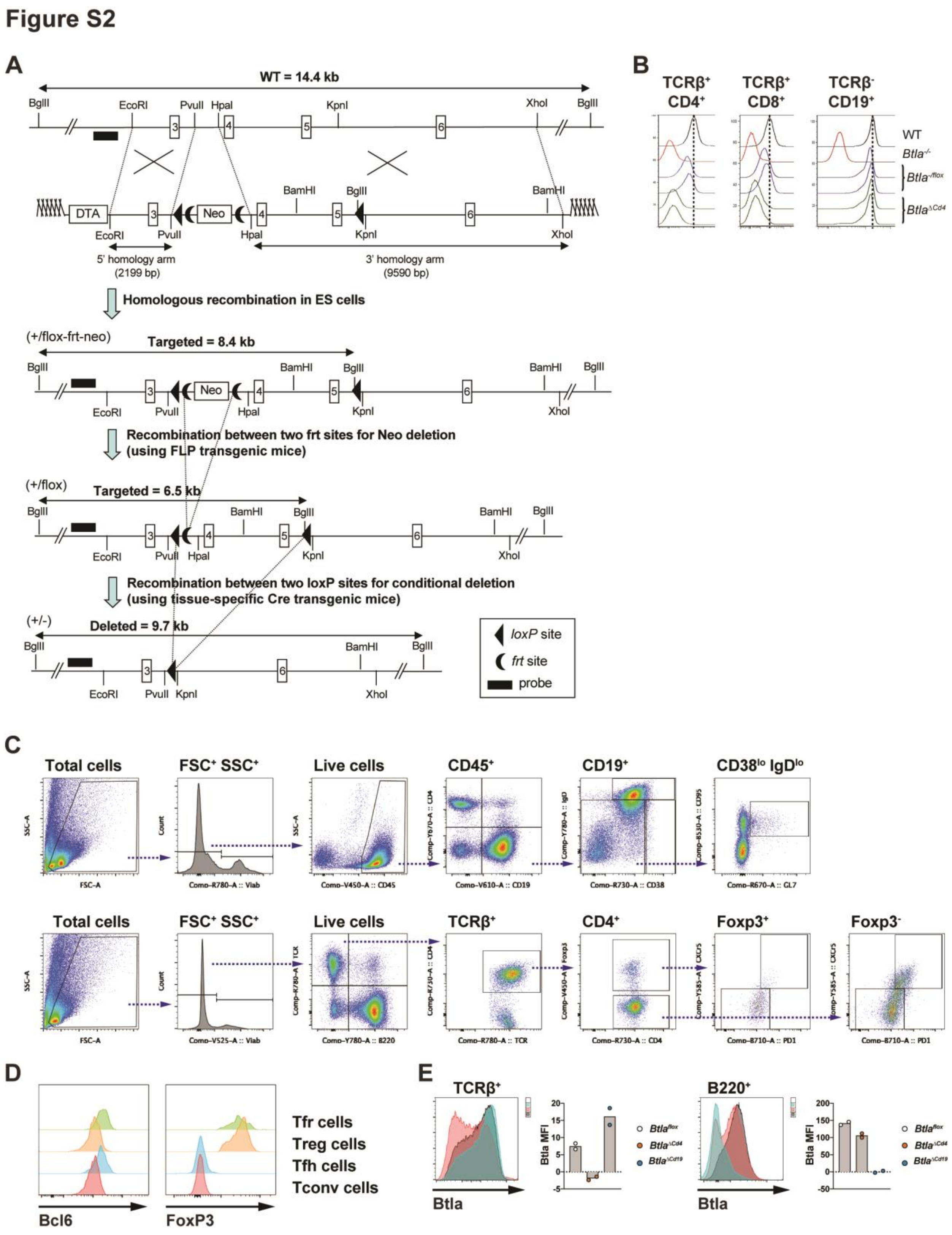

Figure S2. ***Btla* gene targeting and confirmation.** Related to main **Figure 2**, **3**. **A.** Strategy for

Btla gene targeting and germline transmission. The mouse *Btla* genomic locus and the targeted *Btla* gene construct. A frt (moon shape)-flanked neo cassette was inserted into intron 3 of the *Btla* gene by homologous recombination in ES cells. Two loxP sites (triangle) were also inserted 5' and 3' of the frt-neo-frt cassette as indicated. The targeted *Btla* locus with the Southern probe indicated. Mice containing targeted *Btla* locus were bred with FLP animals to delete the frt-neo-frt cassette *in vivo* to obtain neo-less $Btla^{+/flox}$ animals. $Btla^{+/flox}$ animals were bred with tissue-specific Cre transgenic animals to obtain $Btla^{+/-}$ genotype in targeted lineages. **B.** Overlaid histograms show expression of Btla in $CD4^+$ T cells (left), $CD8^+$ T cells (middle), and $CD19^+$ B cells (right) from total splenocytes isolated from wild-type, $Btla^{-/-}$, $Btla^{-/flox}$, and $Btla^{-/\Delta Cd4}$ animals. **C.** Gating strategy used to identify lymphocyte subsets lymphoid tissues. **D.** Overlaid histograms of Bcl6 (left) and Foxp3 (right) staining in Tconv, Tfh, Treg and Tfr cells. **E**. Overlaid histograms of Btla staining in $Tcr\beta^+$ (left) and $B220^+$ (right) cells from $Btla^{flox}$, $Btla^{\Delta Cd4}$, and $Btla^{\Delta Cd19}$ animals. Graphs of Btla MFI are shown at right.

Figure S3

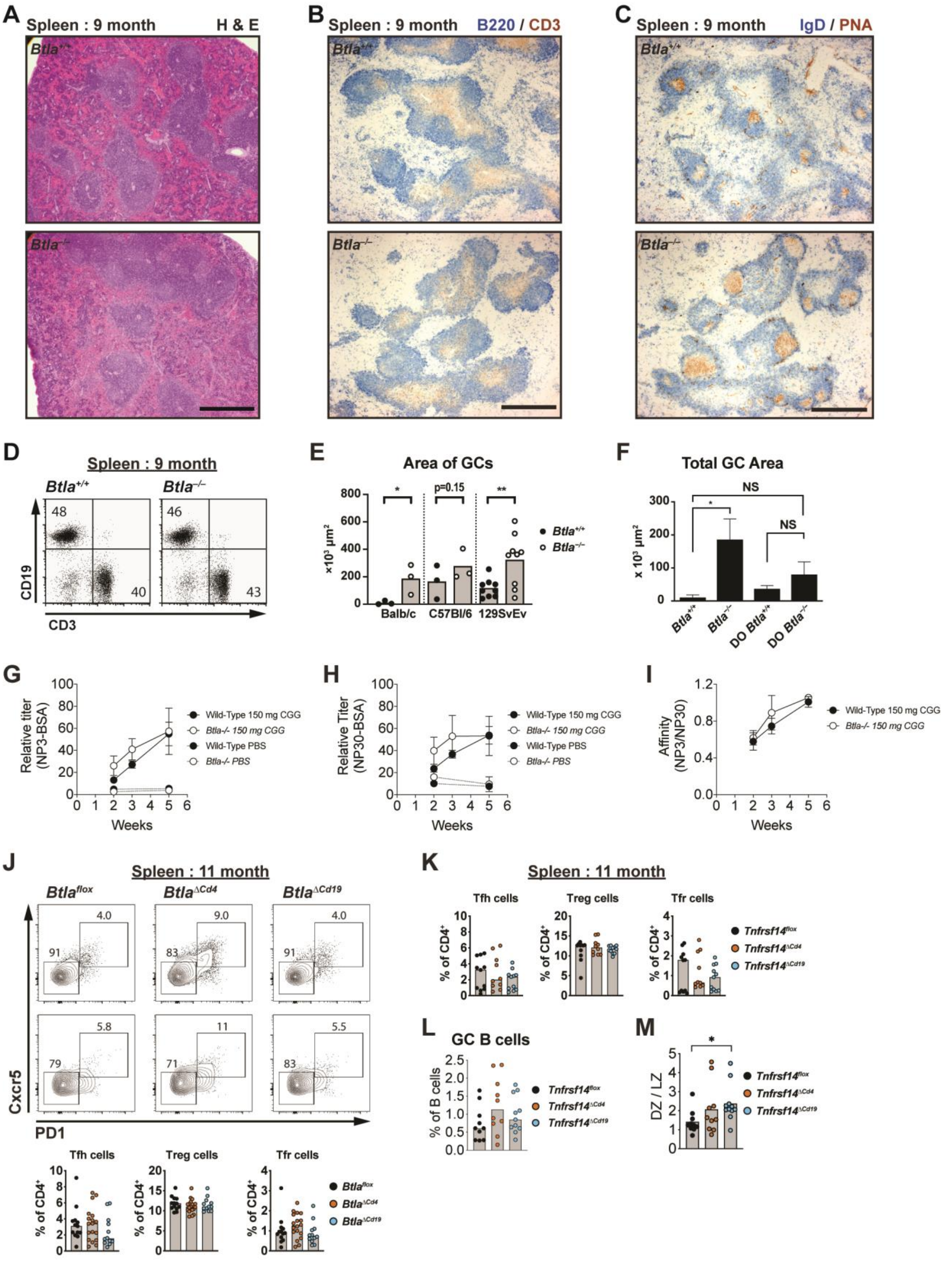
A
Spleen : 9 month
H & E
Btla+/+
Btla−/−
B
Spleen : 9 month
B220 / CD3
Btla+/+
Btla−/−
C
Spleen : 9 month
IgD / PNA
Btla+/+
Btla−/−
D
Spleen : 9 month
Btla+/+
Btla−/−
48
40
46
43
CD19
CD3
E
Area of GCs
p=0.15
×10³ μm²
Btla+/+
Btla−/−
Balb/c
C57Bl/6
129SvEv
F
Total GC Area
NS
x 10³ μm²
Btla+/+
Btla−/−
DO Btla+/+
DO Btla−/−
G
Relative titer (NP3-BSA)
Weeks
Wild-Type 150 mg CGG
Btla-/- 150 mg CGG
Wild-Type PBS
Btla-/- PBS
H
Relative Titer (NP30-BSA)
Weeks
Wild-Type 150 mg CGG
Btla-/- 150 mg CGG
Wild-Type PBS
Btla-/- PBS
I
Affinity (NP3/NP30)
Weeks
Wild-Type 150 mg CGG
Btla-/- 150 mg CGG
J
Spleen : 11 month
Btlaflox
BtlaΔCd4
BtlaΔCd19
4.0
91
9.0
83
4.0
91
5.8
79
11
71
5.5
83
Cxcr5
PD1
Tfh cells
Treg cells
Tfr cells
% of CD4+
Btlaflox
BtlaΔCd4
BtlaΔCd19
K
Spleen : 11 month
Tfh cells
Treg cells
Tfr cells
% of CD4+
Tnfrsf14flox
Tnfrsf14ΔCd4
Tnfrsf14ΔCd19
L
GC B cells
% of B cells
Tnfrsf14flox
Tnfrsf14ΔCd4
Tnfrsf14ΔCd19
M
DZ / LZ
Tnfrsf14flox
Tnfrsf14ΔCd4
Tnfrsf14ΔCd19

Figure S3. Histological analysis and antigen responses of Btla-/- animals. Related to main **Figure 2.**, **3. A.** Paraffin sections from aged wild-type or *Btla*$^{-/-}$ spleens were stained with H&E to visualize splenic architecture. Scale bar equals 500 µm. **B.**, **C.** Frozen serial sections from aged wild-type or *Btla*$^{-/-}$ spleens were stained for B220 and CD3 (**B.**) or IgD and PNA (**C.**). Scale bar equals 500 µm. **D.** Splenocytes from aged wild-type and *Btla*$^{-/-}$ animals were analyzed for the frequency of CD3^{+} and B220^{+} cells. **E.**, **F.** Graphs show the total GC area from spleens collected from wild-type and *Btla*$^{-/-}$ animals backcrossed to the Balb/c, C57BL/6, and 129SvEv strains (**E.**) and backcrossed to the Balb/c and TCR-transgenic DO11.10 strains (**F.**). **G.**-**I**. Graphs show anti-hapten titers to NP3 (**G.**), NP30 (**H.**), and the relative increase in antibody affinity (**I.**) over time in wild-type and *Btla*$^{-/-}$ animals immunized with 150 mg NP-chicken gamma globulin (CGG) measured by ELISA. **J**. Splenocytes from aged *Btla*^{flox}, *Btla*$^{\Delta Cd4}$, and *Btla*$^{\Delta Cd19}$ animals were analyzed by flow cytometry to detect the frequency of T cell subsets. Representative plots from 11-month-old animals show gated CD4^{+}Foxp3^{-} cells and CD4^{+}Foxp3^{+} cells (bottom panels). **K.** The frequency of Tfh, Treg, and Tfr cells within the spleens of aged *Tnfrsf14*^{flox}, *Tnfrsf14*$^{\Delta Cd4}$, and *Tnfrsf14*$^{\Delta Cd19}$ animals was analyzed and plotted. **L.** The frequency of GC B cells within the spleens of aged *Tnfrsf14*^{flox}, *Tnfrsf14*$^{\Delta Cd4}$, and *Tnfrsf14*$^{\Delta Cd19}$ animals was analyzed and plotted. **M.** The ratio of DZ / LZ cells within GC B cells in the spleens of aged *Tnfrsf14*^{flox}, *Tnfrsf14*$^{\Delta Cd4}$, and *Tnfrsf14*$^{\Delta Cd19}$ animals was analyzed and plotted. GC area and titer analysis analyzed using Student's t-test. Cellularity data analyzed using multi-parameter linear modeling ANOVA. Error bars indicates SEM. *****, $p < 0.05$; ****,** $p < 0.01$. Cellularity replicate analysis relates to main figure data. RT-PCR data is representative of two experiments with at least n=4 replicates for each condition.

Figure S4

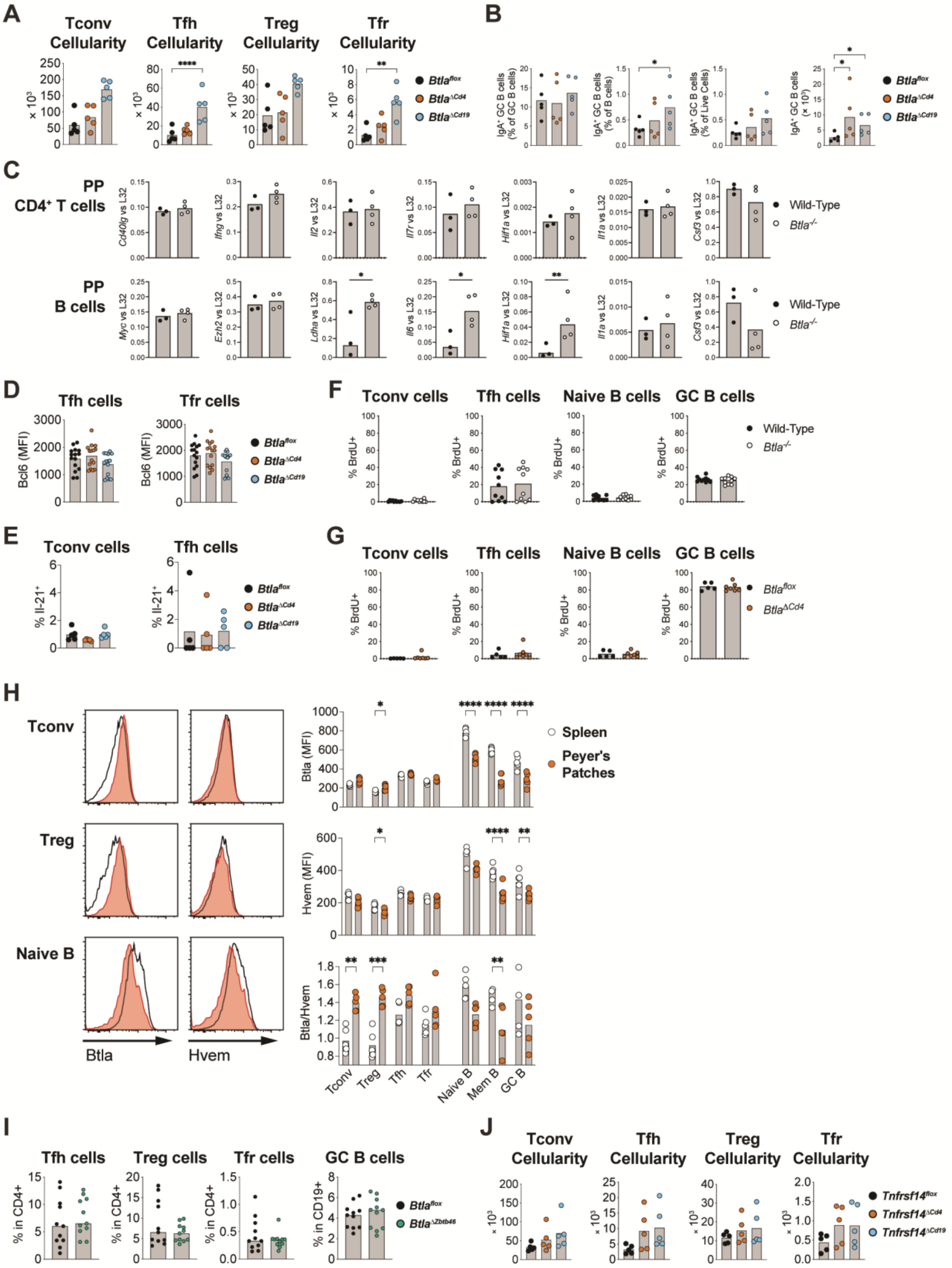

A
Tconv Cellularity
Tfh Cellularity
Treg Cellularity
Tfr Cellularity
B
C
PP CD4⁺ T cells
PP B cells
Wild-Type
D
Tfh cells
Tfr cells
E
Tconv cells
Tfh cells
F
Naive B cells
GC B cells
G
H
Tconv
Treg
Naive B
Btla
Hvem
Spleen
Peyer's Patches
I
Treg cells
Tfr cells
J

Figure S4. Analysis in *Btla*^{flox}, *Btla*$^{\Delta Cd4}$, and *Btla*$^{\Delta Cd19}$ animals of cellularity, gene expression, proliferation and Btla/Hvem ratios. Related to main **Figure 4.**, **5. A.** Graphs of the numbers of Tconv, Tfh, Treg, and Tfr cells in PP in 8-week-old *Btla*^{flox}, *Btla*$^{\Delta Cd4}$, and *Btla*$^{\Delta Cd19}$ animals. **B.** Graphs of IgA^{+} GC B cells shown as a percent of all GC B cells, of total B cells, of total live cells, and of the total cellularity of all IgA^{+} GC B cells from the Peyer's patches of 8-week-old wild-type, *Btla*$^{-/-}$, *Btla*$^{\Delta Cd4}$, and *Btla*$^{\Delta Cd19}$ animals. **C.** Graphs show expression of indicated transcripts in purified CD4^{+} T cells (top) or purified B cells (bottom) isolated from untreated wild-type or *Btla*$^{-/-}$ Peyer's patches. Replicate data analyzed by 2way ANOVA. **D.** Graphs of Bcl6 protein expression in Tfh (left) and Tfr (right) cells in PP in 8-week-old *Btla*^{flox}, *Btla*$^{\Delta Cd4}$, and *Btla*$^{\Delta Cd19}$ animals. **E.** Graphs of Il-21 expression in Tconv (left) and Tfh (right) cells in PP in 8-week-old *Btla*^{flox}, *Btla*$^{\Delta Cd4}$, and *Btla*$^{\Delta Cd19}$ animals. **F.**, **G.** Graphs of the percent BrdU uptake in Tconv, Tfh, Naïve B, and GC B cells cells in PP of 8-week-old wild-type and *Btla*$^{-/-}$ animals (**F.**), or in Tconv, Tfh, Naïve B, and GC B cells cells in PP of 8-week-old *Btla*^{flox} and *Btla*$^{\Delta Cd4}$ animals (**G.**) injected intraperitoneally with 2 mg BrdU. **H.** Overlaid histograms of Btla (right) and Hvem (left) expression in Tconv (top), Treg (middle), and naïve B cells (bottom) from spleen (open) or PP (shaded). Graphs of the expression of Btla (top), Hvem (middle), and the ratio of Btla to Hvem (bottom) in T and B cell subsets is shown. Data analyzed by 2way ANOVA. **I.** Graphs of the frequency of Tfh, Treg, and Tfr cells among CD4^{+} T cells and GC B cells among total B cells in *Btla*^{flox} and *Btla*$^{\Delta Zbtb46}$ animals. **J.** Graphs of the numbers of Tconv, Tfh, Treg, and Tfr cells in PP in 8-week-old *Tnfrsf14*^{flox}, *Tnfrsf14*$^{\Delta Cd4}$, and *Tnfrsf14*$^{\Delta Cd19}$ animals. Data analyzed using multi-parameter linear modeling ANOVA. *****, $p < 0.05$; ****,** $p < 0.01$; ***, $p < 0.001$; ****, $p < 0.0001$. Cellularity replicate analysis relates to main figure data.

Figure S5

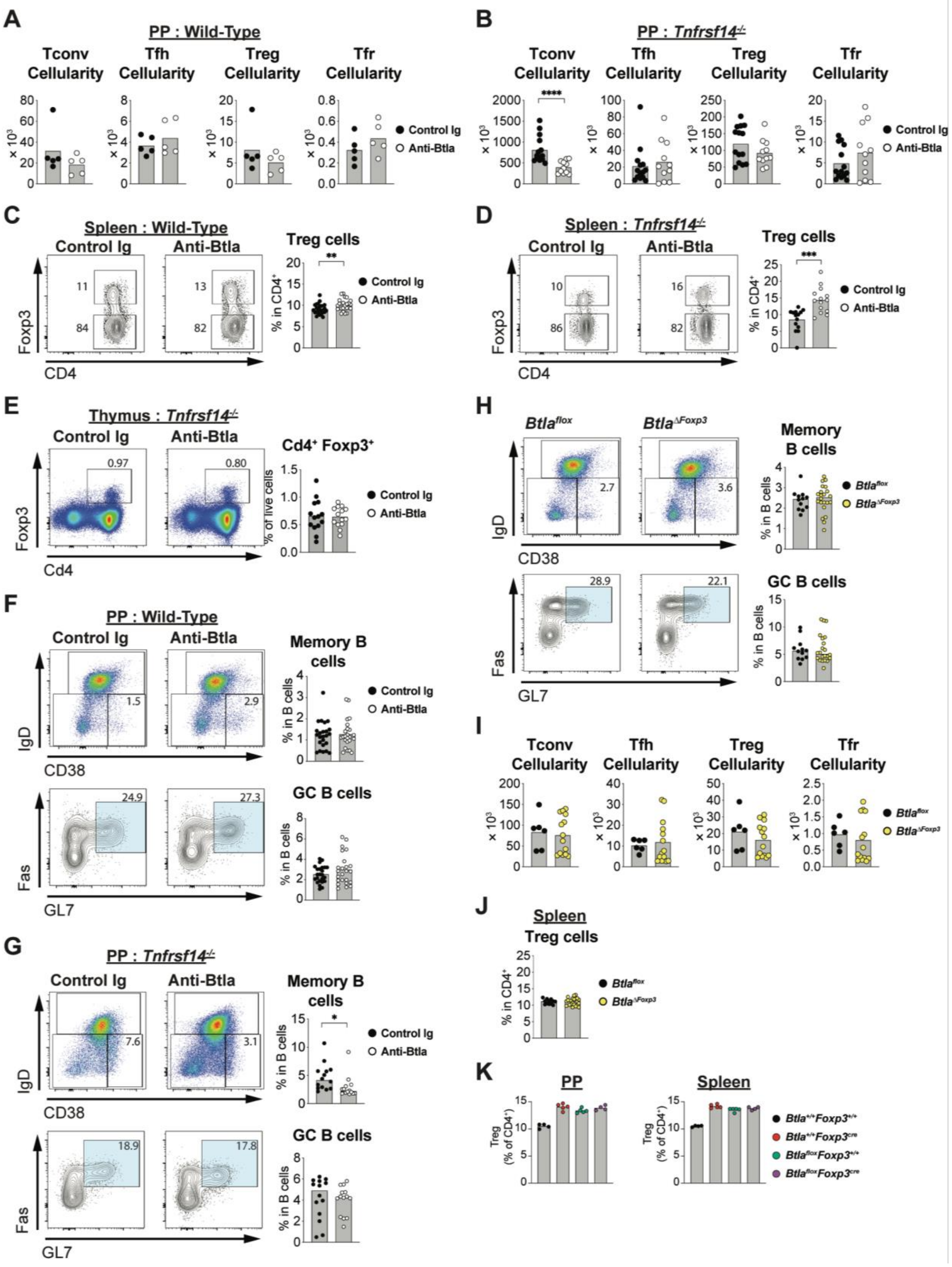

Figure S5. **Characterization Btla regulation of Treg homeostasis.** Related to main **Figure 6.**

A., **B.** Graphs of the numbers of Tconv, Tfh, Treg, and Tfr cells in PP in the PP of 8-week-old wild-type (**A.**) or *Tnfrsf14*$^{-/-}$ (**B.**) animals treated with control Ig or anti-Btla as in **Figure 6**. **C.**, **D.** Analysis of Treg cells from spleens of wild-type (**C.**) or *Tnfrsf14*$^{-/-}$ (**D.**) animals treated with control Ig or anti-Btla as in **Figure 6**. Representative plots of CD4^{+} cells are shown. The frequency of gated CD4^{+}Foxp3^{+} cells is plotted at right. **E.** Analysis of Foxp3 expression in thymocytes of *Tnfrsf14*$^{-/-}$ animals treated with control Ig or anti-Btla as in **Figure 6**. Representative plots of live cells are shown. The frequency of gated CD4^{+}Foxp3^{+} cells is plotted at right. **F.**, **G.** Analysis of B cell subsets from PP of wild-type (**F.**) or *Tnfrsf14*$^{-/-}$ (**G.**) animals treated with control Ig or anti-Btla as in **Figure 6**. Representative plots of total B cells (top) and gated CD38^{low}IgD^{low} B cells (bottom) are shown. The frequency of CD38^{hi}IgD^{low} memory B cells (top) and of GL7^{+}Fas^{+} GC B cells (bottom) is plotted at right. **H.** Analysis of B cell subsets from PP of *Btla*^{flox} and *Btla*$^{\Delta Foxp3}$ animals. Representative plots of total B cells (top) and gated CD38^{low}IgD^{low} B cells (bottom) are shown. Graphs of the frequency of CD38^{hi}IgD^{low} memory B cells and of GL7^{+}Fas^{+} GC B cells is shown at right. **I.** Graphs of the numbers of Tconv, Tfh, Treg, and Tfr cells in PP in 8-week-old *Btla*^{flox} and *Btla*$^{\Delta Foxp3}$ animals. **J.** Analysis of Treg cells from spleens of *Btla*^{flox} and *Btla*$^{\Delta Foxp3}$ animals. Graph show the frequency of gated CD4^{+}Foxp3^{+}PD1^{-}Cxcr5^{-} cells. **K.** Graphs of the percent of FoxP3^{+} Tregs within CD4^{+} cells in PP (left) or spleen (right) of *Btla*$^{+/+}$*Foxp3*$^{+/+}$, *Btla*$^{+/+}$*Foxp3*^{cre}, *Btla*$^{+/flox}$*Foxp3*$^{+/+}$, and *Btla*$^{+/flox}$*Foxp3*^{cre}. Data analyzed using multi-parameter linear modeling ANOVA. *****, p < 0.05; ****,** p < 0.01; ***, p < 0.001; ****, p < 0.0001. Cellularity replicate analyses relates to main figure data.

Figure S6

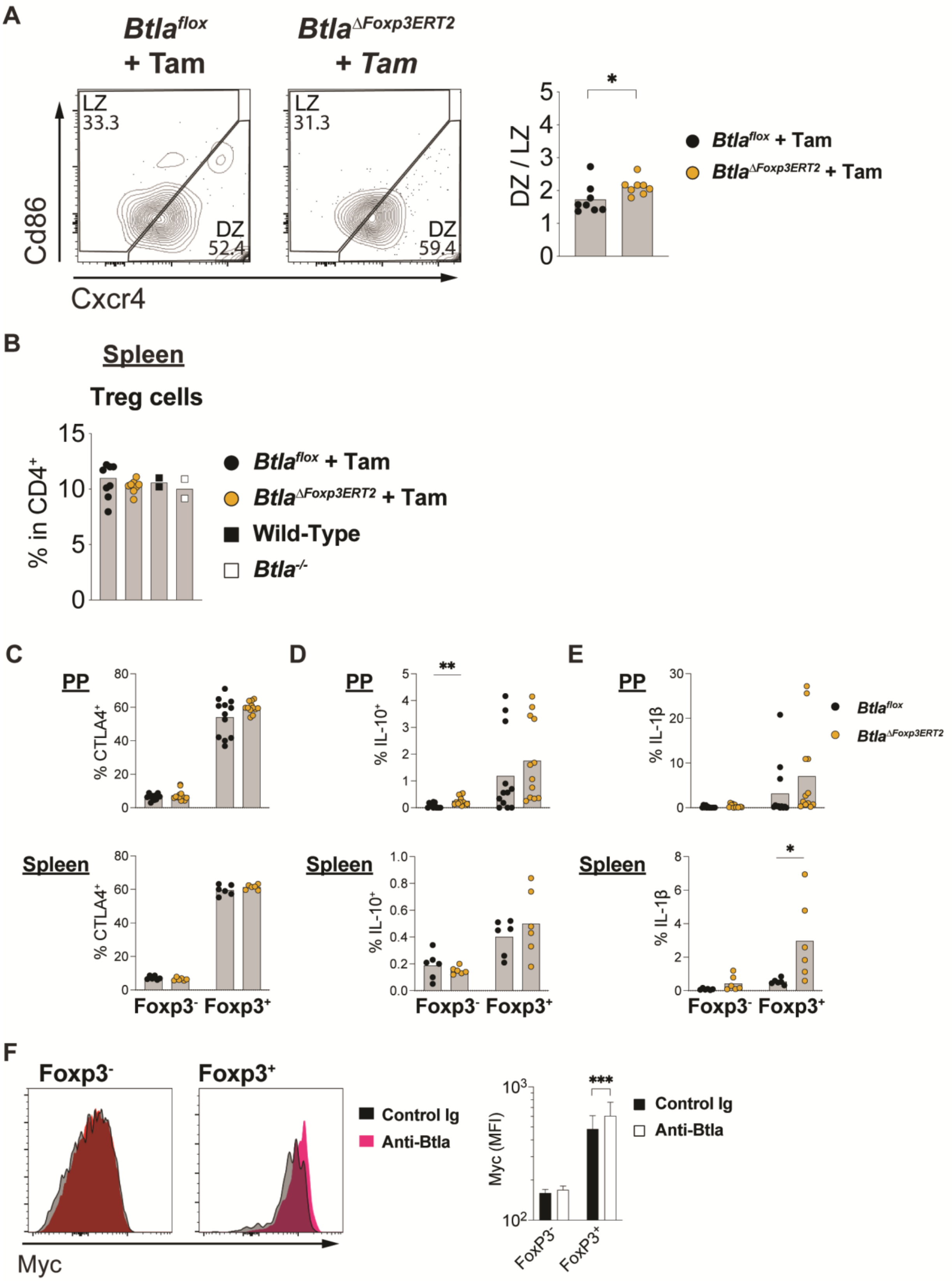

A
Btla^flox + Tam
Btla^ΔFoxp3ERT2 + Tam
LZ 33.3
DZ 52.4
LZ 31.3
DZ 59.4
Cd86
Cxcr4
DZ / LZ
*
Btla^flox + Tam
Btla^ΔFoxp3ERT2 + Tam
B
Spleen
Treg cells
% in CD4+
Btla^flox + Tam
Btla^ΔFoxp3ERT2 + Tam
Wild-Type
Btla-/-
C
PP
Spleen
% CTLA4+
Foxp3- Foxp3+
D
PP
Spleen
% IL-10+
**
Foxp3- Foxp3+
E
PP
Spleen
% IL-1β
*
Btla^flox
Btla^ΔFoxp3ERT2
Foxp3- Foxp3+
F
Foxp3-
Foxp3+
Control Ig
Anti-Btla
Myc
Myc (MFI)
***
FoxP3-
FoxP3+
Control Ig
Anti-Btla

Figure S6. Inducible deletion of Btla in Tregs. Related to main **Figure 6**. **A.** Analysis of GC B cells from PP of *Btla*^{flox} and *Btla*$^{\Delta Foxp3ERT2}$ animals injected intraperitoneally with 1.5 mg tamoxifen over three days as in **Figure 6.** Representative plots show GC B cells to determine the relative abundance of Cxcr4^{hi}CD86^{lo} DZ and Cxcr4^{lo}CD86^{hi} LZ cells. DZ / LZ cell ratio is plotted at right. **B.** Analysis of Treg cells from spleens of untreated wild-type and *Btla*$^{-/-}$ animals, and *Btla*^{flox} and *Btla*$^{\Delta Foxp3ERT2}$ animals injected intraperitoneally with 1.5 mg tamoxifen over three days as in **Figure 6**. Graphs show the frequency of gated CD4^{+}Foxp3^{+}PD1^{-}Cxcr5^{-} cells. *, $p < 0.05$. **C.-E.** Graphs of protein expression of Ctla-4 (**C.**), Il-10 (**D.**), and Il-1β (**E.**) in PP (top) and spleen (bottom) Foxp3^{-} and Foxp3^{+} T cells from tamoxifen treated *Btla*^{flox} and *Btla*$^{\Delta Foxp3ERT2}$ animals as in **Figure 6**. **F.** Representative histogram overlays show the expression of Myc in Foxp3^{-} (left) and Foxp3^{+} (right) CD4^{+} T cells from control Ig and Btla mAb treated SRBC-immunized animals, and graph of Myc protein levels in Foxp3^{-} and Foxp3^{+} CD4^{+} T cells is shown at right. Data analyzed using Student's t-test. Error bars indicates SD. *****, $p < 0.05$; ******,** $p < 0.01$; ***, $p < 0.001$. Cellularity replicate analyses relates to main figure data.

Figure S7

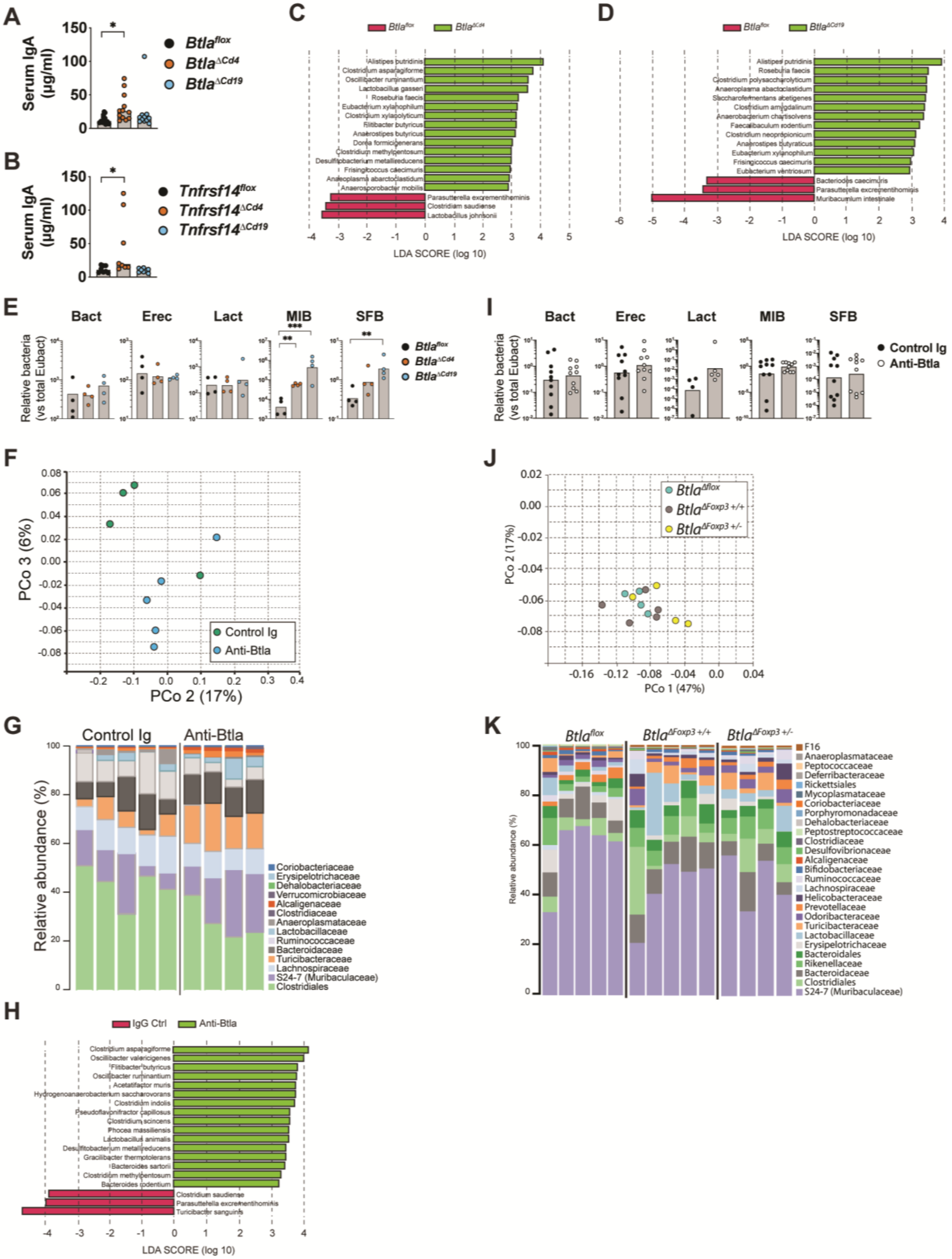

A
Serum IgA (µg/ml)
Btla^flox
Btla^ΔCd4
Btla^ΔCd19
B
Tnfrsf14^flox
Tnfrsf14^ΔCd4
Tnfrsf14^ΔCd19
C
D
LDA SCORE (log 10)
E
Bact
Erec
Lact
MIB
SFB
Relative bacteria (vs total Eubact)
I
Control Ig
Anti-Btla
F
PCo 3 (6%)
PCo 2 (17%)
J
PCo 2 (17%)
PCo 1 (47%)
G
Control Ig
Anti-Btla
Relative abundance (%)
H
IgG Ctrl
Anti-Btla
K
Relative abundance (%)

Figure S7. Btla and Hvem regulation of intestinal microbiota signatures. Related to main **Figure 7**. **A.-B.** Graphs show concentration of serum IgA in 8-week-old *Btla*^{flox}, *Btla*$^{\Delta Cd4}$, and *Btla*$^{\Delta Cd19}$ animals (**A.**), or of *Tnfrsf14*^{flox}, *Tnfrsf14*$^{\Delta Cd4}$, and *Tnfrsf14*$^{\Delta Cd19}$ animals (**B.**) measured by ELISA. **C.-D.** Graphs show LEfSe comparison analysis between *Btla*^{flox} vs *Btla*$^{\Delta Cd4}$ (**C.**) and *Btla*^{flox} vs *Btla*$^{\Delta Cd19}$ (**D.**). **E.** Graphs show the relative abundance of bacterial species compared to total Eubacteria in fecal pellets from *Btla*^{flox}, *Btla*$^{\Delta Cd4}$, and *Btla*$^{\Delta Cd19}$ experimental cohorts measured by QRT-PCR. **F.-I.** PCoA plot of microbial β-diversity (**F.**), graph of relative microbial family abundance (**G.**), graph of LEfSe comparison analysis (**H.**), and the relative abundance of bacterial species compared to total Eubacteria (**I.**) in fecal pellets from control hamster Ig and anti-Btla (clone 6A6) treated animals. **J.**, **K.** PCoA plot of microbial β-diversity (**J.**) and graph of relative microbial family abundance (**K.**) in fecal pellets of *Btla*^{flox}, *Btla*$^{\Delta Foxp3+/+}$, and *Btla*$^{\Delta Foxp3+/-}$ experimental animals. Eubact, *Eubacteria*; Bact, *Bacteroides*; Erect, *Eubacterium rectale*/*Clostridium coccoides*; Lact, *Lactobacillus*/*Lactococcus*; MIB, Mouse intestinal *Bacteroides*; SFB, Segmented filamentous bacteria. Data analyzed using multi-parameter linear modeling ANOVA. *****, p < 0.05; ****,** p < 0.01; ***, p < 0.001. IgA replicate analyses for flox lines relates to main figure data. Unsorted bacterial analyses in antibody treated animals is representative of two experiments with at least n=4 replicates for each condition.

Supplemental Table

Table S1. Oligonucleotide sequences for RT-PCR analysis. Related to **METHODS**. All oligotnucleotide sequences are reported here for dertermination of RNA transcript levels. Additionally oligonucleotide sequences are reported for determination of microbiota abundance.

Primer: Btla forward: GGGAATTCTTCATCCTCCATC	This paper	N/A
Primer: Btla reverse: GTTGCACTGGACACTCTTCATC	This paper	N/A

Primer: Myc forward: ATGCCCCTCAACGTGAACTTC	This paper	N/A
Primer: Myc reverse: CGCAACATAGGATGGAGAGCA	This paper	N/A
Primer: Bcl6 forward: CTGCAGATGGAGCATGTTGT	This paper	N/A
Primer: Bcl6 reverse: GCCATTTCTGCTTCACTGG	This paper	N/A
Primer: Foxo1 forward: CCCAGGCCGGAGTTTAACC	This paper	N/A
Primer: Foxo1 reverse: GTTGCTCATAAAGTCGGTGCT	This paper	N/A
Primer: Irf4 forward: TCCGACAGTGGTTGATCGAC	This paper	N/A
Primer: Irf4 reverse: CCTCACGATTGTAGTCCTGCTT	This paper	N/A
Primer: Irf8 forward: CGGGGCTGATCTGGGAAAAT	This paper	N/A
Primer: Irf8 reverse: CACAGCGTAACCTCGTCTTC	This paper	N/A
Primer: Prdm1 forward: TGCGGAGAGGCTCCACTA	This paper	N/A
Primer: Prdm1 reverse: TGGGTTGCTTTCCGTTTG	This paper	N/A
Primer: Tnfsf13b forward: AACAGACGCGCTTTCCAG	This paper	N/A
Primer: Tnfsf13b reverse: CAGGAGGAGCTGAGAGGTCTAC	This paper	N/A
Primer: Il21 forward: GACATTCATCATTGACCTCGTG	This paper	N/A
Primer: Il21 reverse: TCACAGGAAGGGCATTTAGC	This paper	N/A
Primer: Hif1a forward: ACCTTCATCGGAAACTCCAAAG	This paper	N/A
Primer: Hif1a reverse: CTGTTAGGCTGGGAAAAGTTAGG	This paper	N/A
Primer: Cd40lg forward: CAAGGCGGCAAATACCCAC	This paper	N/A
Primer: Cd40lg reverse: AACACTCCGCCCAAGTGAAC	This paper	N/A
Primer: Ifng forward: ATGAACGCTACACACTGCATC	This paper	N/A
Primer: Ifng reverse: CCATCCTTTTGCCAGTTCCTC	This paper	N/A
Primer: Il2 forward: CAGGATGGAGAATTACAGGAACCT	This paper	N/A
Primer: Il2 reverse: TGGCCTGCTTGGGCAA	This paper	N/A
Primer: Il7r forward: GCGGACGATCACTCCTTCTG	This paper	N/A
Primer: Il7r reverse: AGCCCCACATATTTGAAATTCCA	This paper	N/A
Primer: Il1a forward: CGAAGACTACAGTTCTGCCATT	This paper	N/A
Primer: Il1a reverse: GACGTTTCAGAGGTTCTCAGAG	This paper	N/A
Primer: Csf3 forward: ATGGCTCAACTTTCTGCCCAG	This paper	N/A
Primer: Csf3 reverse: CTGACAGTGACCAGGGGAAC	This paper	N/A
Primer: Ezh2 forward: AGTGACTTGGATTTTCCAGCAC	This paper	N/A
Primer: Ezh2 reverse: AATTCTGTTGTAAGGGCGACC	This paper	N/A
Primer: Ldha forward: TGTCTCCAGCAAAGACTACTGT	This paper	N/A
Primer: Ldha reverse: GACTGTACTTGACAATGTTGGGA	This paper	N/A
Primer: Il6 forward: TAGTCCTTCCTACCCCAATTTCC	This paper	N/A
Primer: Il6 reverse: TTGGTCCTTAGCCACTCCTTC	This paper	N/A
Primer: L32 forward: GGATCTGGCCCTTGAACCTT	This paper	N/A

Primer: L32 reverse: GAAACTGGCGGAAACCCA	This paper	N/A
Primer:V3-V4 Forward: TCGTCGGCAGCGTCAGATGTGTATAAGAGACAGCCTACGGGNGGCWGCAG	This paper	N/A
Primer:V3-V4 Reverse: GTCTCGTGGGCTCGGAGATGTGTATAAGAGACAGGACTACHVGGGTATCTAATCC	This paper	N/A
Total Eubacteria	Barman et al., 2008	N/A
Bacteroides	Barman et al., 2008	N/A
Eubacterium rectale/Clostridium coccoides	Barman et al., 2008	N/A
Lactobacillus/Lactococcus	Barman et al., 2008	N/A
Mouse intestinal Bactoides	Barman et al., 2008	N/A
Segmented filamentous bacteria	Barman et al., 2008	N/A
